# HOXD3 promotes the migration and angiogenesis of hepatocellular carcinoma via modifying hepatocellular carcinoma cells exosome-delivered CCR6 and regulating chromatin conformation of CCL20

**DOI:** 10.1038/s41419-024-06593-x

**Published:** 2024-03-16

**Authors:** Lumin Wang, Chenyang Qiao, Lili Han, Xiaofei Wang, Jiyu Miao, Li Cao, Chen Huang, Jinhai Wang

**Affiliations:** 1 Department of Gastroenterology, The Second Affiliated Hospital of Xi’an Jiaotong University, Xi’an, 710004 P. R. China; 2 Department of Oncology, The Second Affiliated Hospital of Xi’an Jiaotong University, Xi’an, 710004 P. R. China; 3 Key Laboratory of Environment and Genes Related to Diseases, Xi’an Jiaotong University Health Science Center, Xi’an, Shaanxi 710061 P. R. China; 4 Department of Hematology, The Second Affiliated Hospital of Xi’an Jiaotong University, Xi’an, 710004 P. R. China

**Keywords:** Cancer microenvironment, Oncogenes, Tumour angiogenesis

## Abstract

Angiogenesis plays an essential role in the microenvironment of hepatocellular carcinoma (HCC). HOXD3 is involved in the metastasis and invasion of HCC cells; Whereas the underlying molecular mechanisms in the microenvironment of HCC remain unknown. Wound healing, transwell invasion, tube formation and spheroid sprouting assays were carried out to identify the effects of HCC-HOXD3-exosomes and genes on the migration of HCC cells. ChIP–PCR was applied to test the binding region of HOXD3 on CCR6, Med15, and CREBBP promoter. Exosome isolation and mRNA-seq were applied to examine the morphological characteristics of exosomes and the contained mRNA in exosomes. Co-IP and Immunofluorescence assays were used to demonstrate the role of CREBBP in the chromatin conformation of CCL20. The nude mice were used to identify the function of genes in regulating migration of HCC in vivo. In this study, integrated cellular and bioinformatic analyses revealed that HOXD3 targeted the promoter region of CCR6 and induced its transcription. CCR6 was delivered by exosomes to endothelial cells and promoted tumour migration. Overexpression of CCR6 promoted metastasis, invasion in HCCs and angiogenesis in endothelial cells (ECs), whereas its downregulation suppressed these functions. The role of HOXD3 in the metastasis and invasion of HCC cells was reversed after the suppression of CCR6. Furthermore, CCL20 was demonstrated as the ligand of CCR6, and its high expression was found in HCC tissues and cells, which was clinically associated with the poor prognosis of HCC. Mechanistically, HOXD3 targets the promoter regions of CREBBP and Med15, which affect CCL20 chromatin conformation by regulating histone acetylation and expression of Pol II to enhance the migration of HCCs. This study demonstrated the function of the HOXD3–CREBBP/Med15–CCL20–CCR6 axis in regulating invasion and migration in HCC, thus providing new therapeutic targets for HCC.

## Introduction

Hepatocellular carcinoma is one of the most common cancers and the second leading cause of cancer-related deaths [1]. Because of its increasing morbidity and mortality, liver cancer has emerged as a global healthcare concern [2]. However, effective treatment modalities for liver cancer are limited. The preferred treatment strategies, namely, surgical resection, radiotherapy and chemotherapy, impose a risk of disease recurrence, leading to a poor prognosis [3]. In recent years, with the progress of immunology, significant development has been made in immunotherapy for the treatment of hepatocellular carcinoma. However, immunotherapeutic drugs are expensive and have toxic side effects. Therefore, it is important to understand the molecular mechanisms underlying the migration and invasion of HCC and determine their correlation with the regulation of gene expression to improve the diagnosis and treatment of HCC.

Recent studies on the molecular mechanism of HCC migration and invasion have focused not only on the interior of cells but also on the tumour microenvironment (TME). The interplay between malignant and surrounding cells is necessary within TME and can lead to tissue remodelling and angiogenesis within the tumour, resulting in the proliferation, metastasis and invasion of HCC cells [4]. Multiple angiogenesis-related factors and signalling pathways participate in tumour-associated vasculature formation, such as vascular endothelial growth factor (VEGF), chemokines and angiopoietin [5–7]. In our previous study, HOXD3 was found to induce angiogenesis and regulate cell migration and invasion [8].

The HOX family is an evolutionarily conserved family which encodes a homeodomain consisting of 61 amino acids. In vertebrates, 39 HOX family numbers have been identified, which have been arranged tandemly in four similar clusters, namely, HOXA, HOXB, HOXC and HOXD [9]. The subfamily of HOXD transcription factors included HOXD1, HOXD3, HOXD4, HOXD8, HOXD9, HOXD10, HOXD11, HOXD12 and HOXD13. HOX proteins were initially found to play a role in early embryonic development. Accumulating evidence shows that HOX expression is more frequently associated with cancer and is abnormal in many primary tumours. In addition, the HOX genes play a crucial role in regulating the growth, apoptosis, differentiation, adhesion, invasion, migration and angiogenesis of cancer cells [10–12]. The expression of HOXD3, a member of the HOXD subfamily, is markedly increased in numerous cancers, such as HCC [13], gastric cancer (GC) [14] and breast cancer [15]. In our previous study on HCC, MeCP2 combined with CREB1 to promote the expression of HOXD3 by binding to the promoter hypermethylation region of HOXD3 in vivo and in vitro [8]. In addition, YY1 recruits HDAC1 to inhibit the proliferation of HCC cells by targeting the promoter region of HOXD3 through the ITGA2–ERK1/2 cell signalling [16]. Whereas, the regulatory role of HOXD3 in the TME of HCC remains unclear, and molecular mechanisms underlying HOXD3-mediated angiogenesis within the TME of HCC warrant further investigation.

Among different factors that participated in tumour angiogenesis, the chemokine superfamily is one of the most important factors. Members of this family contain a great many proteins, which perform various biological functions by binding to their receptors [17]. CCL20 and its receptor CCR6 are involved in the progression, migration, invasion and angiogenesis of cancer cells [18–20], including HCC cells [21]. In colorectal cancer, CCR6 enhances tumour angiogenesis via the AKT/NF-κB/VEGF cell signalling. Upregulation of CCL20 is connected with metastasis and poor prognosis in HCC [22]. The CCL20–CCR6 contributes to the angiogenesis of HCC, which is induced by the hepatitis C virus (HCV). Therefore, CCL20/CCR6 signalling plays a crucial role in the proliferation and metastasis of HCC. However, the relationship between HOXD3 and CCL20/CCR6 in HCC remains unknown.

In this study, HOXD3 was found to target the promoter region of CCR6 and induce its transcription. CCR6 was transported to endothelial cells by HCC-HOXD3-exosomes and promoted ECs angiogenesis. In addition, HOXD3 targeted the promoter regions of CREBBP and Med15, which affected the chromatin conformation of CCL20 by regulating histone acetylation and expression of RNA polymerase II (Pol II). Therefore, we speculated that HOXD3 affects the angiogenesis of ECs and enhances the metastasis and invasion of HCC cells by modulating CCL20 and transporting CCR6. The effect of the HOXD3–CREBBP/Med15–CCL20–CCR6 axis on HCC migration and angiogenesis was further investigated, which could clarify a novel mechanism of action of HOXD3 in hepatocellular carcinoma.

## Results

### CCR6 is highly higher expressed in exosomes of HOXD3-induced HCC cells

**Fig. 1:**
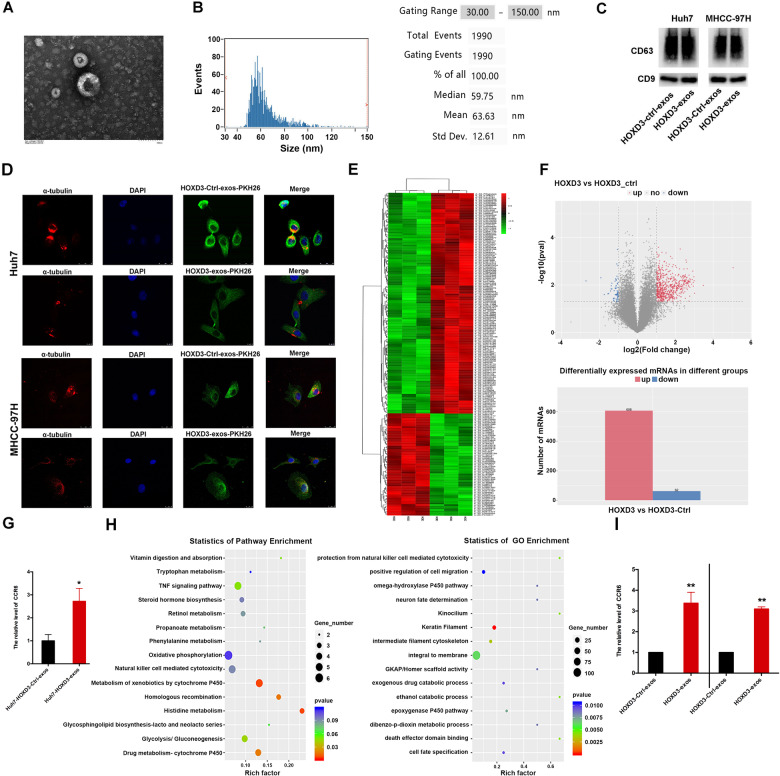
CCR6 is secreted by HCC cells in the pattern of exosomes. **A** The image of exosomes under transmission electron microscopy. **B** Nanoparticle tracking analysis was used to determine the absolute exosome size distribution and concentration of exosomes. **C** Western blotting analysis of the expression of exosomal markers (CD63, CD9) in HOXD3-Ctrl and HOXD3 treated HCC cell supernatants. **D** Fluorescence microscopic images of PKH26-labelled exosomes incubated with Huh7 and MHCC-97H cells. Huh7-HOXD3-Ctrl-exosomes, Huh7-HOXD3-exosomes, MHCC-97H-HOXD3-Ctrl-exosomes and MHCC-97H-HOXD3-exosomes were added into Huh7 and MHCC-97H cells, respectively. **E**, **F** The exosomes-mRNA-seq was used to identify the expression of genes in Huh7-HOXD3-Ctrl-exosomes and Huh7-HOXD3-exosomes. **G** mRNA-seq showed that CCR6 mRNA level in Huh7-HOXD3-exosomes, Huh7-HOXD3-Ctrl-exosomes was the negative control. **H** The statistics of pathway and GO enrichment in exos-mRNA-seq. **I** qRT-PCR analysis of CCR6 levels in exosomes purified from HOXD3-Ctrl and HOXD3 treated HCC cell supernatants(*, *P* < 0.05; **, *P* < 0.01).

Exosomes derived from Huh7 and MHCC-97H cells transfected with HOXD3 and its negative control, HOXD3-Ctrl, were isolated from the supernatants using sequential differential centrifugation. The morphological characteristics of the exosomes were analysed using a transmission electron microscope (TEM), revealing cup-and round-shaped exosomes with well-defined membrane structures (Fig. 1A). The predominant size of the exosomes was determined to be 63 nm (Fig. 1B). Furthermore, western blotting results confirmed the presence of exosomal markers CD63 and CD9 in exosomes derived from both HOXD3-Ctrl- and HOXD3-transfected Huh7 and MHCC-97H cells (Fig. 1C). Flow cytometry analysis using a cytoflex instrument showed that exosomes labelled with CD63 and CD81 accounted for 58.76% and 52.23% of the total exosomes in MHCC-97H-HOXD3-Ctrl-exosomes, 59.57% and 53.79% of the total exosomes in MHCC-97H-HOXD3-exosomes, 55.15% and 57.78% of the total exosomes in Huh7-HOXD3-Ctrl-exosomes, and 57.09% and 57.77% of the total exosomes in Huh7-HOXD3-exosomes (Fig. S1). Collectively, these findings suggest that exosomes induced by HOXD3 in Huh7 and MHCC-97H cells possess typical exosomal features. These exosomes were utilised for subsequent experiments.

An immunofluorescence assay was conducted to detect the presence of exosomes in HCC cells. DAPI and PKH26 were employed to label the cell nucleus and exosomes, respectively. After 24 h, microscopic analysis confirmed the successful uptake of PKH26-labelled exosomes by HCC cells (Fig. 1D). To examine the content of these exosomes, mRNA-seq was performed (Fig. 1E). Comparative analysis between Huh7-HOXD3-Ctrl-exosomes and Huh7-HOXD3-exosomes revealed 608 upregulated and 62 downregulated genes (Fig. 1F), including increased expression of CCR6 (Fig. 1G, *P* < 0.05). The TNF and other signalling pathways were found to be involved in the regulation of exosomes (Fig. 1H). To validate these findings, the relative expression of CCR6 was assessed through qRT-PCR. Significantly higher expression of CCR6 was observed in HCC-HOXD3-exosomes compared to HCC-HOXD3-Ctrl-exosomes (Fig. 1I, *P* < 0.01). These findings indicate that HCC cells internalise exosomes and that exosomes treated with HOXD3 in HCC cells exhibit high expression of CCR6.

### HOXD3-induced HCC cell-derived exosomal CCR6 enhances metastasis, invasion and angiogenesis in vitro

**Fig. 2:**
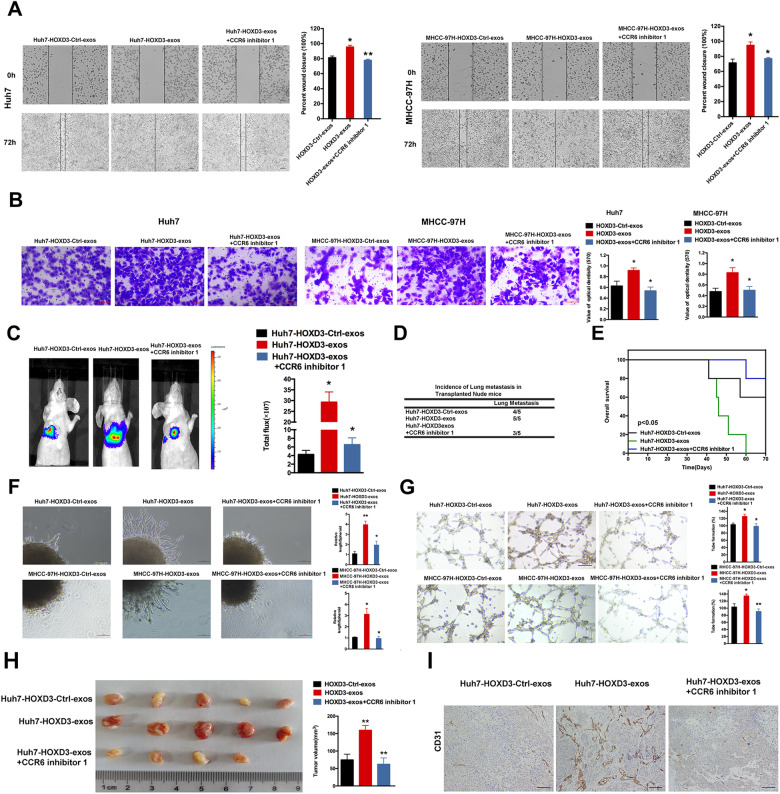
HCC-HOXD3-exosomes induced metastasis, invasion and angiogenesis in vitro by delivering CCR6. **A**, **B** Cell migration and transwell invasion assays were performed to identify cell ability of metastasis and invasion, respectively, and quantitative analysis is shown in graphs on the right (scale bar = 100 µm) (*, *P* < 0.05, *P* < 0.01). **C** Bioluminescence images of nude mice. **D** The liver metastasis incidence. **E** Overall survival of the indicated groups of nude mice. **F** Representative images of spheroid sprouting (scale bar = 100 µm). **G** HCC-HOXD3-exosomes stimulated tube formation in HUVECs (scale bar = 100 µm). **H** Representative images of harvested matrigel plugs are shown (Left). The volume of the tumour (Right). **I** The plugs were immunostained with anti-CD31 antibodies.

A scratch wound healing assay was employed to investigate the involvement of HOXD3-induced exosomes from HCC cells in metastasis. Compared with HCC-HOXD3-Ctrl-exosomes, HCC-HOXD3-exosomes had a high degree of motility in both HCC cell lines (Fig. 2A). Furthermore, the results of the invasion assay revealed that the HCC cell invasion in the HCC-HOXD3-exosome was higher than that of cells in the HCC-HOXD3-Ctrl-exosome (Fig. 2B). To further elucidate the contribution of CCR6 to migration and invasion, a CCR6 inhibitor (CCR6 inhibitor 1) was introduced to MHCC-97H and Huh7 cells. The results revealed that inhibition of CCR6 attenuated the promoting effects of HCC-HOXD3-exosomes on HCC cell migration and invasion (Fig. 2A, B). In vivo metastasis assay further confirmed that HCC-HOXD3-exosomes enhanced bioluminescent imaging (BLI) signals (Fig. 2C), increased liver metastasis incidence (Fig. 2D), and reduced overall survival in the HCC-HOXD3-exosome group (Fig. 2E). However, inhibition of CCR6 decreased the incidence of lung migration and induced to increase of overall survival in the Huh7-HOXD3-exosomes+CCR6 inhibitor-1 group (Fig. 2C–E).

In addition, spheroids consisting of HCC-HOXD3-Ctrl-exosome- or HCC-HOXD3-exosome-treated HUVECs were generated to assess the sprouting capacity of endothelial cells. In line with the results of the cell migration and transwell invasion analysis, HCC-HOXD3-exosomes enhanced the length of newly developed sprouts (Fig. 2F). Furthermore, a tube formation assay was carried out to evaluate the role of HCC-HOXD3-exosomes in the angiogenesis of HUVECs. The data demonstrated that the number of tubular structures formed in HCC-HOXD3-exosome-treated HUVECs was significantly increased (Fig. 2G). Meantime, inhibition of CCR6 counteracted the promoting role of HCC-HOXD3-exosomes in the angiogenesis of HCC cells (Fig. 2F, G). To further verify the role of exosomes in angiogenesis, a matrigel plug assay was used to detect the newly formed blood vessels in the transplanted gel plugs in nude mice. These results revealed that the plugs containing exosomes derived from HOXD3-treated HCCs markedly enhanced the relative tube length when compared with the HCC-Ctrl transfected HCC cells. Remarkably, exosomes also increased tumour size (Fig. 2H). Inhibiting the expression of CCR6 could reverse the effect of exosomes on HCCs. Additionally, the CD31-positive area was significantly higher in Huh7-HOXD3-exosomes than in Huh7-HOXD3-Ctrl-exosomes, but these changes were mitigated when CCR6 was inhibited (Fig. 2I). Therefore, CCR6-enhanced metastasis and invasion of HCCs, as well as angiogenesis of ECs, are facilitated through HOXD3-induced HCC cell-derived exosomes.

To further examine the connection between HOXD3 and CCR6, the HCCs-shHOXD3 + CCR6-exos and HCCs-shHOXD3-exos were introduced to MHCC-97H and Huh7 cells. The results revealed that the upregulation of CCR6 mitigated the inhibitory effects of HCC-shHOXD3-exosomes on the migration and invasion of HCC cells and the angiogenesis of ECs (Fig. S2A–D). Meanwhile, compared to the HCCs-shHOXD3-Ctrl-exos and HCCs-shCCR6-Ctrl-exos, the suppression of HOXD3 and CCR6 expression inhibited the biological functions of HCCs. In HCCs treated with shHOXD3, the inhibition of CCR6 enhanced the suppressive effects on HCC migration and invasion. Moreover, the wound healing transwell, tube formation and spheroid sprouting assays indicated a significant increase in the number of invading or migrating cells and the sprouting capacity in HCCs-HOXD3-exos or HCCs-CCR6-exos compared to the HCCs-control-exos (Fig. S3A–D).

### CCR6 promotes migration, invasion and angiogenesis of HCCs and ECs in vitro

**Fig. 3:**
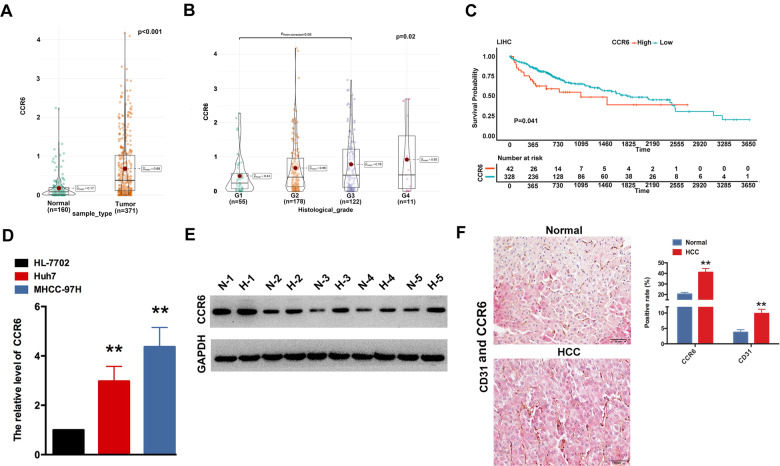
CCR6 is upregulated in HCC. **A** TCGA and GTEx databases were carried out to evaluate CCR6 expression in HCC and healthy tissues (*P* < 0.001). **B** Correlation between the expression of CCR6 and the histological grade of HCC (*P* = 0.02). **C** High CCR6 expression was connected with shorter survival in patients with HCC (*P* = 0.041). **D** Analysis of the mRNA expression of CCR6 in Huh7 and MHCC-97H cells, and HL-7702 cells as the control. (**, *P* < 0.01). **E** Protein expression of CCR6 was examined via western blotting. **F** Protein expression of CCR6 (Red) and CD31 (Brown) in hepatocellular carcinoma tissues versus normal tissues.

Data retrieved from TCGA and GTEX revealed significantly higher expression levels of the CCR6 in various tumour tissues compared with normal ones at the mRNA level (Fig. S4), especially in HCC (*p* < 0.001) (Fig. 3A). Clinical significance of CCR6 assays showed that CCR6 positively connected with histological grade in HCC patients (*p* = 0.02) (Fig. 3B). Meantime, the expression of CCR6 induced a lower overall survival rate of the clinical samples (*p* = 0.041) (Fig. 3C). Subsequently, the expression of CCR6 in HCCs was explored. Our data demonstrated that the mRNA levels of CCR6 were elevated in Huh7 and MHCC-97H, compared with HL-7702 (Fig. 3D). Consistently, data retrieved from the western blotting suggested that the expression of CCR6 was dramatically enhanced in HCC samples compared with that of normal tissues (Fig. 3E). CD31 is a marker of angiogenesis, and its expression was identified using immunohistological staining. The results showed that microvessel density (MVD) in CCR6 high-expressed HCC samples was higher than that in normal tissues (Fig. 3F). Those results strongly suggested that CCR6 is considered to be a tumour activator in HCC.

**Fig. 4:**
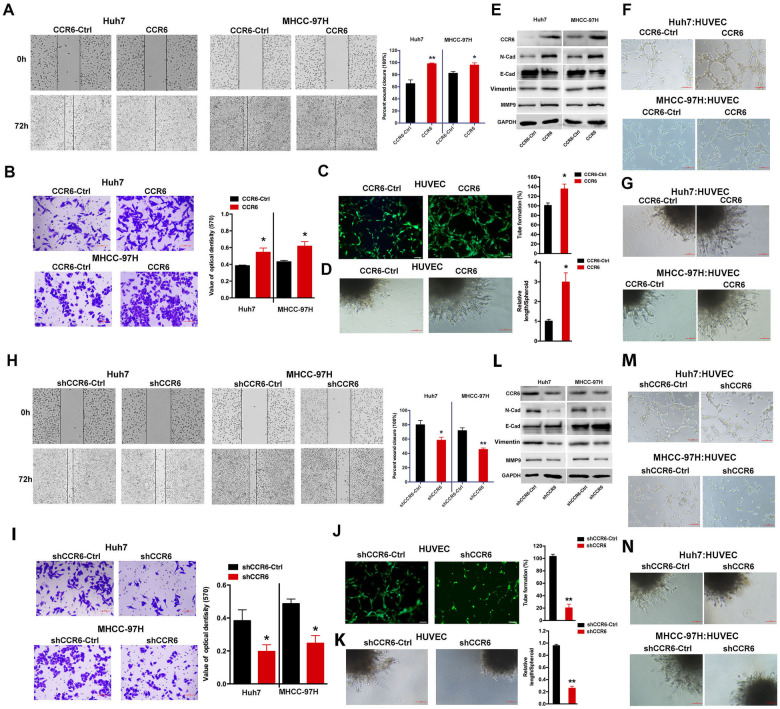
CCR6 enhances the metastasis, invasion and angiogenesis capability of HCCs and ECs. **A**, **B** Scratch wound healing and transwell assays were performed to identify the metastasis and invasion of HCC cells (scale bar = 100 µm) (*, *P* < 0.05; **, *P* < 0.01). **C** Capillary tube formation assay in HUVECs treated with CCR6 and the negative control. **D** Representative images of spheroid sprouting assay are shown (scale bar = 100 µm). **E** HCC cells were transfected with CCR6-Ctrl and CCR6 for 48 h, and then the protein expression of CCR6, N-cadherin, E-cadherin, Vimentin and MMP9 was determined using western blotting. **F**, **G** Tube formation and spheroid sprouting assays were used to test the function of CCR6 on angiogenesis of HUVEC cells in co-cultured HCC cells with HUVEC cells. **H**–**K** Cell migration, transwell invasion, tube formation and spheroid sprouting assays were carried out to demonstrate the effects of CCR6 inhibition on the migration, invasion and angiogenesis capability of HCC cells and EC cells (scale bar = 100 µm) (*, *P* < 0.05; **, *P* < 0.01). **L** The expression of CCR6, MMP9, E-cadherin, Vimentin and N-cadherin at the protein level in HCC cells transfected with shCCR6 and shCCR6-Ctrl. **M**, **N** Tube formation and spheroid sprouting assays were used to demonstrate the role of inhibition of CCR6 on angiogenesis of HUVEC cells in co-cultured HCC cells with HUVEC cells.

Gain- and-loss-of-function assays were performed to examine the effect of CCR6 in HCC. A scratch wound healing assay was performed to highlight the significant role of CCR6 in HCC cell migration. The results showed a higher wound closure rate in CCR6-transfected HCC cells compared to CCR6-Ctrl cells at 48 h and 72 h, indicating that CCR6 enhances the metastasis of Huh7 and MHCC-97H cells (Fig. 4A and Fig. S5). Similarly, compared with the negative control cells, the invasion ability of Huh7 and MHCC-97H cells transfected with CCR6 was increased (Fig. 4B). The results of the tube formation assay revealed that CCR6 induced the formation of capillary-like structures in HUVECs (Fig. 4C). In addition, spheroid sprouting assay revealed that CCR6 increased vessel sprouting (Fig. 4D), supporting its potential role as an enhancer of angiogenesis. Western blotting analysis demonstrated that CCR6 upregulated the expressions of migration-related genes N-cadherin, Vimentin and MMP9 while downregulating the expression of E-cadherin (Fig. 4E). Additionally, a co-culture assay was employed to confirm the influence of CCR6 on HUVEC angiogenesis. The findings demonstrated that CCR6 induced angiogenesis in HUVECs (Fig. 4F, G). However, blocking the activation of CCR6 expression led to a decrease in cell migration, cell invasion, tube formation and the length of newly developed sprouts (Fig. 4H, K). The expression of MMP9, Vimentin and N-cadherin was suppressed, while the expression of E-cadherin increased upon transfection with shCCR6, compared to shCCR6-cCtrl (Fig. 4L). Furthermore, co-culturing HUVECs with shCCR6-treated HCC cells resulted in the suppression of HUVEC angiogenesis (Fig. 4M, N). These findings provide evidence that CCR6 plays a critical role in tumour metastasis and angiogenesis.

### HOXD3 binds to the promoter region of CCR6 to activate its expression

**Fig. 5:**
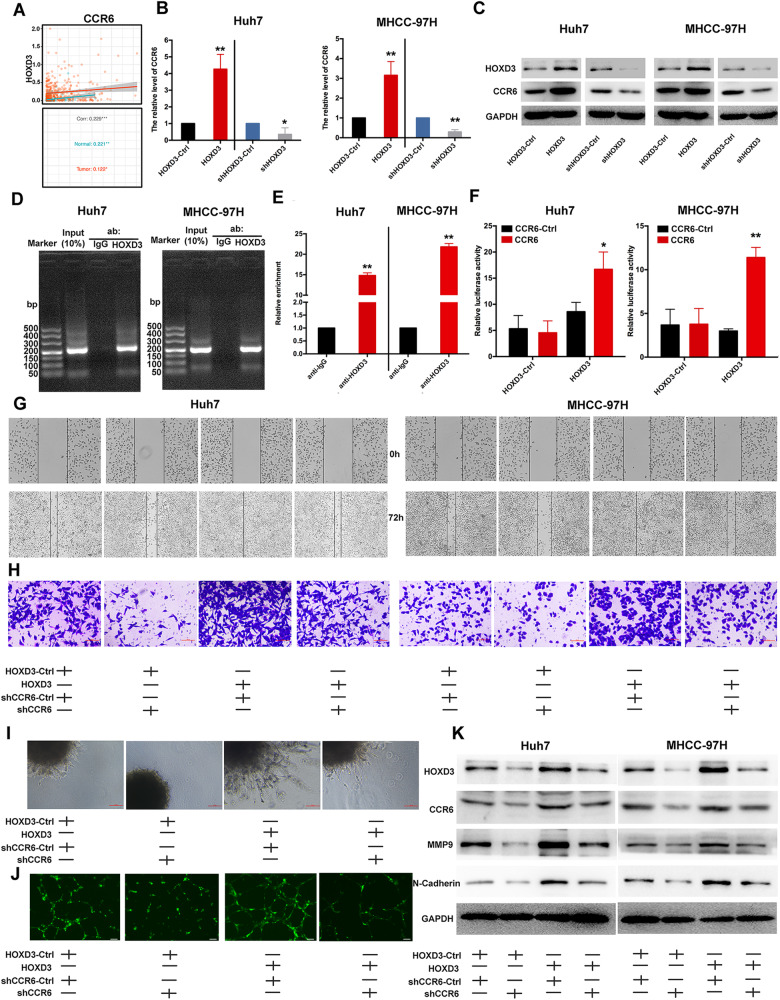
Inhibition of CCR6 reverses the stimulatory function of HOXD3 in the metastasis, invasion and angiogenesis capability of HCC cells and EC cells. **A** TCGA database was performed to examine the correction between CCR6 and HOXD3 in HCC. **B**, **C** The expression of CCR6 was examined at mRNA and protein levels in HCC cells transfected with HOXD3-Ctrl, HOXD3, shHOXD3-Ctrl and shHOXD3 (*, *P* < 0.05; **, *P* < 0.01). **D**–**F** ChIP-PCR and dual-luciferase reporter assays were used to examine the relationship between HOXD3 and CCR6. (*, *P* < 0.05; **, *P* < 0.01). **G**–**J** Wound healing, transwell invasion, tube formation and 3D spheroid sprouting assays revealed that CCR6 downregulation reversed the stimulatory role of HOXD3 in the metastasis, invasion and angiogenesis capability of HCC cells and HUVEC cells (scale bar = 100 µm). **K** Western blotting was performed to examine the HOXD3, CCR6, N-Cadherin and MMP9 expression in HCC cells, which were co-treated with HOXD3 and shCCR6-Ctrl or shCCR6.

qRT-PCR and western blotting were used to examine the association between HOXD3 and CCR6. HOXD3 induced CCR6 expression in HCC cells, whereas inhibition of HOXD3 decreased the expression of CCR6 at the mRNA and protein levels (Fig. 5B, C), which was in line with the data retrieved from the TCGA database (*P* < 0.001, *r* = 0.229) (Fig. 5A). The transcription factor-binding site of HOXD3 in the CCR6 promoter region was predicted to be at 0.6 kb using the UCSC Genome Browser, JASPAR and PROMO database. ChIP assay showed that HOXD3 was specifically associated with the CCR6 promoter (Fig. 5D, E). To explore the effect of HOXD3 on CCR6 transcription, a dual-luciferase reporter assay was performed. Human CCR6 (from −780 to −648) was cloned into pGL3 plasmids. These reporter plasmids were transiently co-transfected with HOXD3-Ctrl/HOXD3 plasmids into Huh7 and MHCC-97H cells separately to assess CCR6 transcriptional activity. The results confirmed that HOXD3 enhanced the transcriptional activity of CCR6 in HCC cells (Fig. 5F). Furthermore, co-transfection of HCC cells with HOXD3 and shCCR6 was conducted to examine the correlation between CCR6 and HOXD3 in HCC cells. These findings revealed that HOXD3 overexpression significantly increased HCC cell migration, invasion capability and HUVEC angiogenesis. However, inhibition of CCR6 markedly counteracted the biological effects induced by HOXD3 in HCC cells (Fig. 5G–J). Additionally, the expression of CCR6, MMP9 and N-cadherin was upregulated in HCC cells with higher HOXD3 expression, but these changes were mitigated when CCR6 was suppressed (Fig. 5K). Overall, these results suggest that HOXD3 promotes metastasis, invasion and angiogenesis by directly modulating CCR6 transcription in HCC cells and ECs.

### HOXD3 regulates the expression of CCL20

**Fig. 6:**
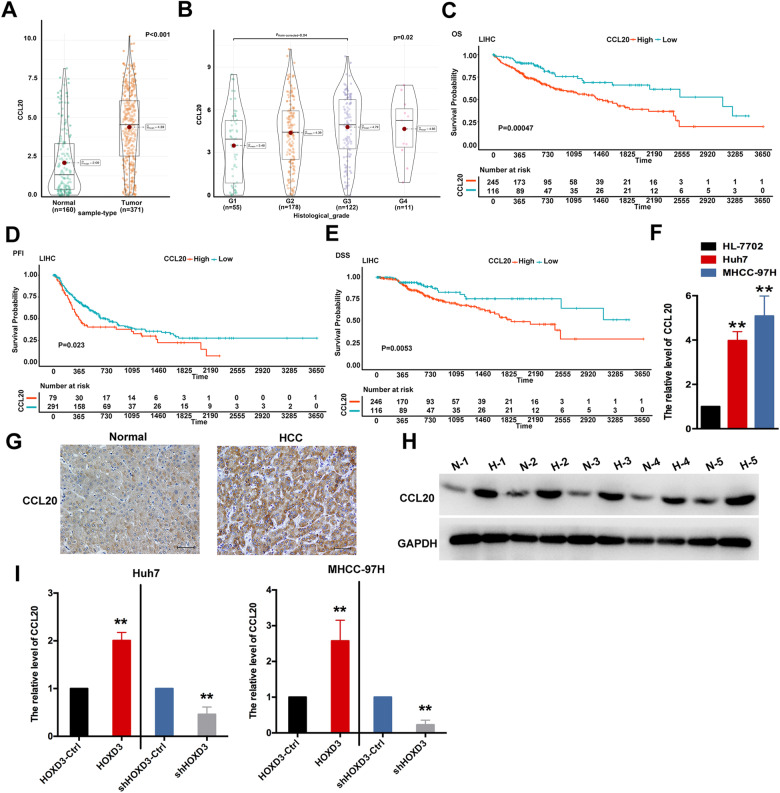
HOXD3 regulates the expression of CCL20. **A** CCL20 expression in 160 normal control tissues and 371 HCC tissues based on TCGA and GTEx databases (*P* < 0.001). **B** Correlation of CCL20 expression with the histological grade of HCC (*P* = 0.02). **C**–**E** Survival curves of OS, PFI and DSS in the high- and low-CCL20 expression groups. **C** P = 0.00047; **D**, *P* = 0.023; **E** *P* = 0.0053. **F** mRNA expression of CCL20 in MHCC-97H and Huh7 cells, HL-7702 cells as the control (**, *P* < 0.01). **G**, **H** The expression of CCL20 at the protein level was examined via IHC analysis and western blotting. **I** The expression of CCL20 at mRNA level in HCC cells treated with HOXD3-Ctrl, HOXD3, shHOXD3-Ctrl and shHOXD3 (**, *P* < 0.01).

TCGA and GTEx databases were performed to examine the association between CCL20 and the carcinogenesis and metastasis of HCC. Data from both databases evaluated that the expression of CCL20 was increased in various cancer tissues (Fig. S6), including HCC, than in normal tissues (*P* < 0.001) (Fig. 6A), and the expression of CCL20 was positively connected with the G grade ((*P* = 0.002) (Fig. 6B). In addition, CCL20 expression was correlated with lower survival rates, including OS (*P* = 0.00047) (Fig. 6C), progression-free interval (PFI; *P* = 0.023) (Fig. 6D) and disease-specific survival (DSS; *P* = 0.0053) (Fig. 6E). The expression of CCL20 at mRNA and protein levels in human HCC cells and tissues were examined via qRT-PCR and IHC analysis. CCL20 expression was found to be higher in Huh7 and MHCC-97H cells than in HL-7702 cells (Fig. 6F). Compared with normal tissues, CCL20 was enhanced in HCC tissues (Fig. 6G, H). Moreover, the expression of CCL20 at mRNA levels was high in HOXD3-treated HCC cells (Fig. 6I). MHCC-97H and Huh7 cells were co-transfected with HOXD3 and shCCL20 to evaluate the correlation between CCL20 and HOXD3 in HCC cells further. Those data revealed that decreased CCL20 markedly suppressed HOXD3-induced promotion of the migration and invasion of HCC cells and angiogenesis of ECs (Fig. S7A–D), suggesting that HOXD3 promoted metastasis and invasion by modulating CCL20 in HCC cells.

### CCL20 enhances migration, invasion and angiogenesis in HCC and ECs in vitro and in vivo

**Fig. 7:**
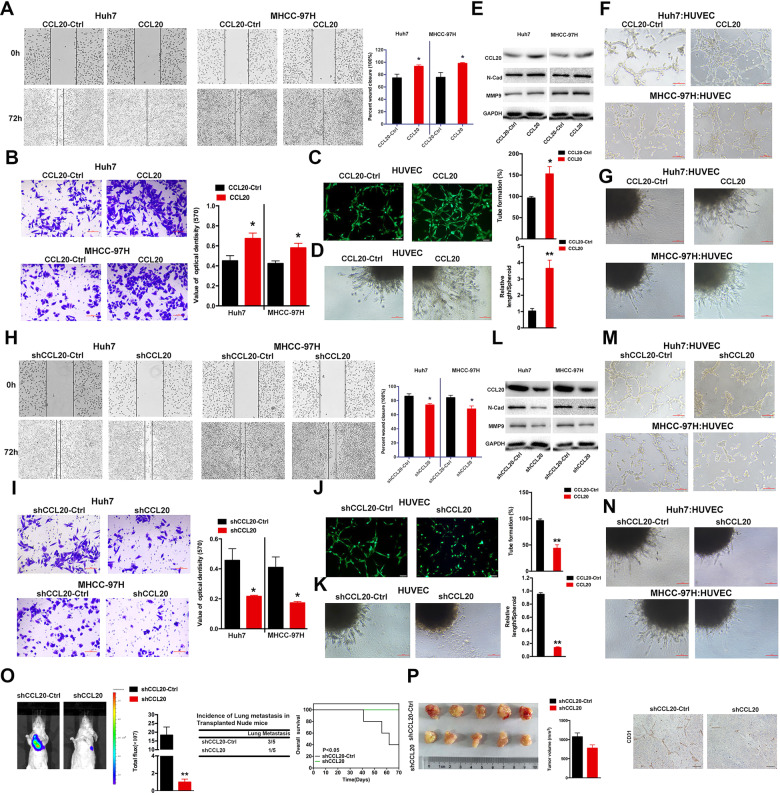
Overexpression of CCL20 promotes cell metastasis, invasion and angiogenesis in HCCs and ECs. **A** Wound healing assay was performed to examine the role of CCL20 overexpression in the migration of HCC cells (scale bar = 100 µm) (*, *P* < 0.05). **B** Effects of high expressed CCL20 on the migration of HCC cells (scale bar = 100 µm) (*, *P* < 0.05). **C** Representative images of tube formation assay. **D** Representative images of spheroid sprouting in HCC cells treated with CCL20-Ctrl and CCL20 (scale bar = 100 µm). **E** Expression of CCL20, N-Cadherin and MMP9 in HCC cells transfected with CCL20-Ctrl and CCL20. **F**, **G** Tube formation and spheroid sprouting assays were applied to verify the function of CCL20 on angiogenesis of HUVEC cells in co-cultured HCC cells with HUVEC cells. **H**–**K** Wound healing, transwell migration, tube formation and spheroid sprouting assays were performed to demonstrate the effects of CCL20 inhibition on the migration, invasion and angiogenesis capability of HCC cells and HUVEC cells (scale bar = 100 µm) (*, *P* < 0.05; **, *P* < 0.01). **L** Protein expression of CCL20, N-cadherin and MMP9 in HCC cells treated with shCCL20 and shCCL20-Ctrl. **M**, **N** Tube formation and spheroid sprouting assays were used to demonstrate the role of inhibition of CCL20 on the angiogenesis of HUVEC cells in co-cultured HCC cells with HUVEC cells. **O** Bioluminescence images and overall survival of the indicated groups of nude mice are shown. **P** Representative images of the harvested matrigel plugs are shown (Left). The volume of tumour (Middle). The plugs were immunostained with anti-CD31 antibodies(Right).

Given its importance in the clinical outcomes of HCC, the function of CCL20 on metastasis, invasion and angiogenesis was further evaluated. The scratch wound healing and transwell invasion assays indicated that CCL20 treatment increased the metastasis ability and motility of cells (Fig. 7A, B). Tube formation assay revealed that CCL20 significantly induced tube formation in endothelial cells (Fig. 7C), and spheroid sprouting assay revealed that CCL20 increased the total sprout length (Fig. 7D). Western blotting was carried out to detect the expression of CCL20 at the protein level. Gain-of-function assay revealed that the N-cadherin and MMP9 expressions were increased in CCL20-transfected HCC cells (Fig. 7E). Meanwhile, co-cultured CCL20-treated HCC with HUVEC were used to verify the function of CCL20 on the angiogenesis of HUVECs. The result showed that CCL20 could lead to an increase in tube formation and spheroid sprouting (Fig. 7F, G). However, the loss of CCL20 decreased the metastasis, invasion and angiogenesis capabilities of HCCs and ECs (Fig. 7H–K). Meanwhile, N-cadherin and MMP9 expression were decreased in shCCL20-transfected HCC cells, compared with shCCL20-Ctrl-transfected HCCs (Fig. 7L). The co-cultured assay further demonstrated that inhibiting CCL20 suppressed the angiogenesis of HUVECs (Fig. 7M, N). Additionally, in vivo, tumour migration and matrigel plug assays in nude mice revealed that CCL20 played a role in regulating tumour migration and angiogenesis. Downregulating CCL20 decreased the incidence of liver migration, resulting in improved overall survival in the shCCL20 group (Fig. 7O). The matrigel plug assay showed that plugs with shCCL20 inhibited the relative tube length compared to shCCL20-Ctrl, and there was also a decreasing trend in tumour volume in the shCCL20 group. Immunostaining for CD31 demonstrated that MVD was reduced in shCCL20-transfected HCC cells compared to the negative control cells (Fig. 7P). These results strongly indicate that CCL20 acts as a tumour activator, contributing to the promotion of HCC metastasis, invasion and EC angiogenesis.

### HOXD3 binds to the promoter region of CREBBP and Med15 to affect the chromatin conformation of CCL20

Given that HOXD3 regulates CCL20 expression, the underlying regulatory mechanisms were further examined. CREBBP and Med15 were found to be involved in CCL20 activation. Clinically, the expression of CREBBP was remarkably enhanced in various cancer tissues (Fig. S8), especially in HCC, compared with that in normal tissues (*P* < 0.001) (Fig. S9A) and resulted in lower OS (*P* = 0.009) (Fig. S9B), PFI (*P* = 0.00082) (Fig. S9C) and disease-free-interval (DFI; *P* = 0.0048) (Fig. S9D) in HCC. Med15, a member of the mediator complex, is participates in tumorigenesis and contributes to the diagnosis, prognosis and treatment of many cancers [23, 24]. Data from TCGA and GTEx revealed that the expression of Med15 was increased in many cancer tissues (Fig. S10), including HCC tissues, compared with that in normal tissues (Fig. S9E). High Med15 expression was remarkably correlated with advanced clinical stage and histological grade (Fig. S9F, G). The OS rates were notably lower among patients with high expressed Med15 than among those with low expressed Med15 (*P* < 0.0001; Fig. S9H). Similarly, the PFI (*P* < 0.001), DFI (*P* = 0.00048) and DSS (*P* = 0.00042) rates were remarkably lower among patients with high expressed Med15 than among those with low expressed Med15 (Fig. S9I–K).

**Fig. 8:**
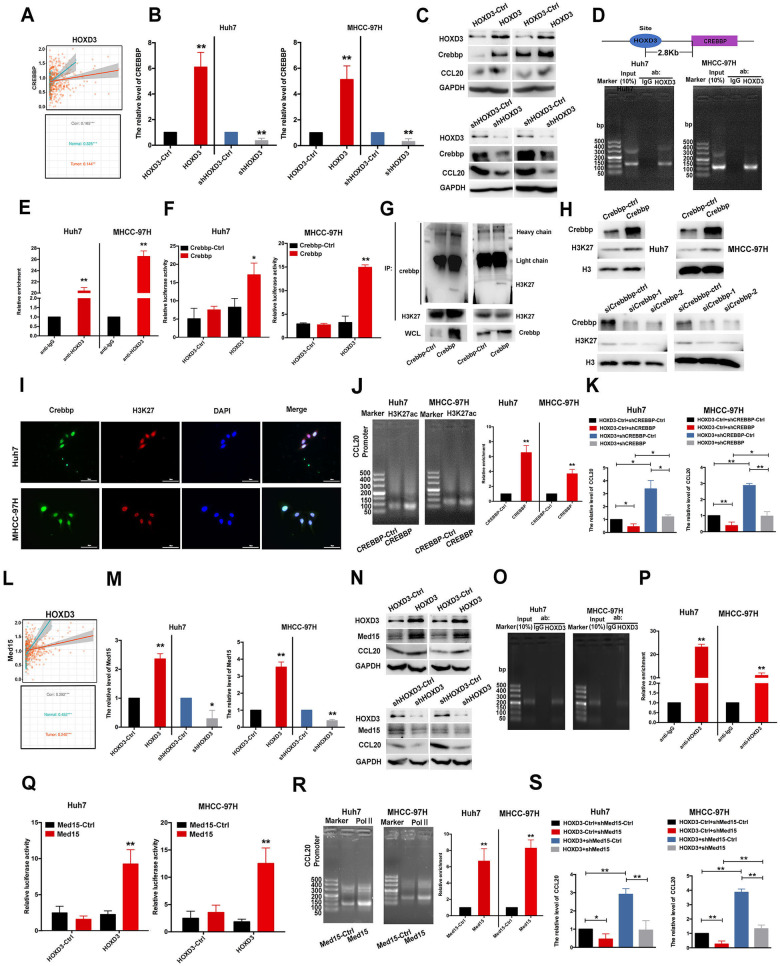
HOXD3 targets the promoter region of CREBBP and MED15, thereby activating CCL20. **A** TCGA database was performed to identify the connection between HOXD3 and CREBBP. **B** After being treated with HOXD3, shHOXD3 and their negative control HOXD3-Ctrl, shHOXD3-Ctrl, respectively. The expression of CREBBP at the mRNA level in HCC cells was detected by qRT-PCR (**P < 0.01). **C** After treated with HOXD3, shHOXD3 and their negative control HOXD3-Ctrl, shHOXD3-Ctrl, respectively. The expression of CREBBP and CCL20 at the protein level in HCC cells was detected by Western blotting. **D**–**F** The connection between HOXD3 and CREBBP was demonstrated using ChIP-PCR and dual-luciferase reporter assays (*, *P* < 0.05; **P < 0.01). **G** Endogenous interaction between CREBBP and H3K27ac in HCC cells. **H** The protein expressions of H3 and H3K27ac in HCC cells after transfected with CREBBP-Ctrl, CREBBP, siCREBBP-Ctrl, siCREBBP-1 and siCREBBP-2. **I** The co-localisation of CREBBP (green) and H3K27ac (red) was assessed by immunofluorescence staining. **J** The role of CREBBP in the regulation of CCL20 via H3K27 modification. **K** RT-PCR was performed to examine the CCL20 expression in HCC cells, which were co-treated with HOXD3 and shCREBBP-Ctrl or shCREBBP (*, *P* < 0.05; **P < 0.01). **L** The correlation between HOXD3 and MED15 was predicted via the TCGA database. **M** The mRNA expressions of MED15 in HCC cells were detected after transfected with HOXD3, shHOXD3 and their negative control HOXD3-Ctrl, shHOXD3-Ctrl, respectively (*, *P* < 0.05; **P < 0.01). **N** The protein expressions of MED15 and CCL20 in HCC cells were detected after transfected with HOXD3, shHOXD3 and their negative control HOXD3-Ctrl, shHOXD3-Ctrl, respectively. **O**–**Q** The transcription factor-binding site of HOXD3 in the MED15 promoter was identified by CHIP-PCR and dual-luciferase reporter assays (**P < 0.01). **R** CHIP assays identified the role of MED15 in the regulation of CCL20 via Pol II. **S** The expression of CCL20 in HCC cells which were co-treated with HOXD3 and shMed15-Ctrl or shMed15 (*, *P* < 0.05; **P < 0.01).

The relationship between HOXD3 and CREBBP was examined to analyse the role of CREBBP in HOXD3-regulated the expression of CCL20. The results revealed that the expression of CREBBP was positively associated with the expression of HOXD3 (Fig. 8A and S11). Consistent with the results of bioinformatic analysis, HOXD3 induced the expression of CREBBP at the mRNA and protein levels in HCC cells (Fig. 8B, C). To verify the correlation between CREBBP and HOXD3, a HOXD3-binding site located 2.8 kb upstream of the CREBBP gene was identified by using the JASPAR, UCSC Genome Browser database and ChIP–PCR assay (Fig. 8D, E). Subsequently, a fragment of CREBBP (−3190 to −2830) was cloned and inserted into the pGL3 vector, and dual-luciferase reporter assay revealed significant enhancement of the luciferase activity of the CREBBP promoter following HOXD3 overexpression (Fig. 8F). CREBBP is involved in histone modification and interacts with a number of transcription factors to modulate transcription of genes via acetylation of histone [25]. As shown in Fig. 8H, the expression of H3K27ac at the protein level in Huh7 and MHCC-97H cells was remarkably distinguishable from that of control cells after either CREBBP overexpression or CREBBP knockdown. Furthermore, the results of the Co-IP analysis verified the protein relationship between CREBBP and H3K27ac in HCC cells (Fig. 8G). This finding was further validated via immunofluorescence staining, which revealed the co-localisation of CREBBP and H3K27ac in HCC cells (Fig. 8I). The biological relationship between CREBBP and CCL20 was examined via ChIP–PCR assay, which revealed a high correlation between H3K27ac and CCL20 in CREBBP-overexpressed HCC cells (Fig. 8J). Inhibition of CREBBP could eliminate the promoting effect of HOXD3 on CCL20 (Fig. 8K). So, HOXD3 bound to the promoter region of CREBBP, which in turn regulated H3K27ac to activate CCL20 expression.

An important connection was demonstrated between HOXD3 and Med15 in HCC by using the database (Fig. 8L and S11). Consistent with the results from the bioinformatic analysis, HOXD3 was found to upregulate the expression of Med15 and CCL20 in HCC cells (Fig. 8M–N) and directly bind to the promoter region of Med15 (Fig. 8O, P). To identify the core activating region, a dual-luciferase reporter assay was performed by co-transfecting the reporter fragment containing the binding site (−503 to −304) with either HOXD3-Ctrl or HOXD3 into HCC cells. The results confirmed the essentiality of this region for the HOXD3-mediated transcriptional activation of Med15 (Fig. 8Q). A previous study showed that Med15 regulates Pol II [26]. In this study, the results of ChIP–PCR assays demonstrated that Med15 induced the combination of Pol II and the promoter region of CCL20 (Fig. 8R). Meanwhile, suppression of Med15 could eliminate the promoting effect of HOXD3 on CCL20 (Fig. 8S), suggesting that HOXD3 enhanced the expression of Med15, which increased CCL20 expression.

## Discussion

Migration is the crucial cause of death among patients with cancer. Angiogenesis is one of the crucial steps in the progression and migration of tumours. In our previous study, we demonstrated that HOXD3 was upregulated in HCC and promoted tumour metastasis by inducing the expression of EGFR and ITGA2 in HCC cells and tissues, indicating that HOXD3 is a crucial angiogenic factor in HCC [8, 16]. Meanwhile, tumour cells secrete various growth factors, including CCL20, which can bind to its specific receptor, CCR6, to accelerate the development of cancer by inducing angiogenesis. The progression, invasion and metastasis of cancer induce dynamic alterations in host tissues, resulting in the development of a mixed TME [27]. Similarly, exosomes, as a part of TME, participate in tumour migration by regulating the immune response and epithelial–mesenchymal transition (EMT) and promoting angiogenesis [28]. However, the effects of HOXD3–CCL20/CCR6 on angiogenesis in HCC, especially in TME, remain unknown. Therefore, in the present study, we demonstrated the function of the HOXD3–CCL20-CCR6 axis in regulating metastasis, migration in HCCs and angiogenesis in ECs and found that HOXD3 mediated the crosstalk between HCC and endothelial cells in TME. Therefore, we speculate that HOXD3 could act as a prospective therapeutic target.

Exosomes are important for intercellular communication and can deliver microRNA-, mRNA- and protein-based transcription factors to various cell types. Recent studies have indicated that miRNAs, mRNAs and lncRNAs participate in tumour progression, migration, and invasion in HCC. Exosomal miR-15a derived from MSCs can suppress the expression of SALL4 and delay HCC development [29]. Exosomal lncRNA SNHG16 enhances metastasis of HCC by modulating the miR-942-3p- MMP9 in Telocytes [30]. Within the tumour microenvironment, HCCs and ECs engage in reciprocal communication via exosomes, influencing the dynamics of angiogenesis, as well as the proliferation, migration and invasion of HCCs [31]. Matsuura et al. investigated the impact of exosomes derived from HCCs on HUVEC tube formation and angiogenic processes. Their research revealed that under hypoxic conditions, HIF-1α upregulates miR-155 expression, leading to the inhibition of ELK3, an ETS transcription factor that suppresses angiogenesis via inhibition of ETS-1 on MT1-MMP [32]. Huang et al. demonstrated that HCC-derived exosomal circ-100338 promotes HCC migration by increasing proangiogenic activity in ECs [33]. Notably, CircCMTM3 acts as a sponge for miR-3619-5p, modulating HUVEC angiogenesis through SOX9 and creating a supportive microenvironment for HCC progression [34]. This study showed that exosomes derived from HOXD3-treated HCC cells (Huh7 and MHCC-97H) enhanced migration and invasion of HCCs. Furthermore, using the assays of 3D spheroid sprouting, and tube formation, the results showed that exosomal derived from HOXD3-treated HCC cells increased the angiogenesis of ECs (Fig. 2). These results were also verified in nude mouse models. Meantime, several studies have reported that exosomes can be used for mRNA delivery in the treatment of multiple diseases [35]. In this study, 608 genes were found to be upregulated, including CCR6 and 62 genes were found to be downregulated at the mRNA level in Huh7-HOXD3-exosomes compared with Huh7-HOXD3-Ctrl-exosomes. In addition, these genes were enriched in many cell signalling pathways, such as those connected with cytokine–cytokine receptor interaction, cell adhesion molecules (CAMs), and the AMPK signalling pathway. Our result showed that CCR6 inhibitor 1 attenuated the role of HCC-HOXD3-exosomes in HCC (Fig. 2). Additionally, we observed that exosomal CCR6 derived from HOXD3-induced HCC cells enhances HCC metastasis, invasion and EC angiogenesis (Fig. S3). Based on these findings, we speculate that CCR6 promotes EC angiogenesis through HCC-HOXD3-mediated exosome transport, subsequently influencing HCC migration and invasion.

Furthermore, CCR6 staining was significantly stronger in tumour tissues and was positively correlated with CD31 staining. High-expressed CCR6 was connected with the histological grade and poor OS of patients with HCC (Fig. 3). Endothelial cell angiogenesis is involved in the invasion and migration of tumour cells. In this study, CCR6 enhanced the invasion, metastasis of HCCs and tube formation of HUVECs. Zhu et al. also demonstrated the influence of CCR6 on the angiogenesis of ECs. They observed that the culture medium of CCR6-overexpressing RKO cells, compared to the control (Ctrl)-RKO, increased the number of intersections and the total length of tubules in HUVECs. Mechanistically, it was found that CCR6 activation in RKO cells activated the AKT/NF-κB pathway, thereby leading to an increase in the secretion of VEGF-A and promoting angiogenesis in neighbouring ECs [20]. Additionally, results showed that CCR6 expression was positively connected with HOXD3 in human HCC samples. HOXD3 could bind to the promoter region of CCR6 to induce its transcription, whereas CCR6 knockdown abrogated HOXD3-enhanced HCC metastasis and invasion (Fig. 5). Therefore, HOXD3 can facilitate HCC metastasis by upregulating CCR6 expression, and CCR6 co-ordinates the tumour progression by playing the multiple functions on HCC and ECs.

CCR6, the sole receptor of CCL20, plays a regulatory role in CCL20-induced tumour migration [36]. The findings of this study revealed that CCL20 is capable of binding to CCR6 in Huh7 and MHCC-97H cells (Fig. S12). CCL20 is a member of the C–C motif chemokine subfamily. According to the structural differences, chemokines are divided into C-C motif (CC), C–X–C motif (CXC), C-motif (C) and CXC3 and have a molecular weight of 8–14 kDa [37]. Many studies have shown that highly expressed CCL20 participates in the progression and migration of numerous cancers [38, 39], including HCC [40]. A recent study reported that CCL20 induces the recruitment of both regulatory T cells (Tregs) and human leucocyte antigen-DR isotype (HLA-DRlo) type 2 conventional dendritic cells (cDC2) to hypoxic TME [41]. In this study, bioinformatic and IHC analyses revealed that CCL20 was aberrantly elevated in tumour tissues compared with the normal tissues, and highly expressed CCL20 resulted in low OS, PFI and DSS rates (Fig. 6). These clinical observations strongly suggest that CCL20 drives the progression and metastasis of HCC. In line with the results of previous studies, these results demonstrated that CCL20 promoted the metastasis, invasion of HCCs and angiogenesis capability of ECs. These results were verified in vivo, wherein suppression of CCL20 inhibited the metastasis of HCC cells (Fig. 7). Furthermore, a positive connection was observed between HOXD3 and CCL20 in HCC cells, and HOXD3 was found to target the promoter regions of CREBBP and Med15. Therefore, we speculated that HOXD3-mediated CREBBP and Med15-induced CCL20 expression increase the migration and invasion capability of HCC cells. CREBBP and Med15 are involved in the remodelling of the chromatin structure and transcriptional regulation of genes.

To verify our hypothesis, we examined the relationship among HOXD3, CREBBP and Med 15 via bioinformatic analyses. Considerable differences were revealed in OS, PFI, DSS, and DFI between the high- and low-expression of the three genes (Fig. S13). Compared with the co-suppression of the three genes, their elevated co-expression resulted in shorter OS. Furthermore, data from TCGA database revealed that the expression of CREBBP was enhanced in HCC tissues, compared with that in normal samples, and was positively correlated with HOXD3 (Figs. S8 and S11). Furthermore, a putative HOXD3-binding motif was predicted in the promoter region of CREBBP using the JASPAR database and UCSC Genome Browser, and the results of ChIP–PCR and dual-luciferase reporter assays verified the binding of endogenous HOXD3 to the abovementioned motif in HCC cells. High and low expressed HOXD3 induced and suppressed CREBBP expression, respectively, at the mRNA and protein levels. CREBBP is a widely expressed transcriptional coactivator and a major lysine acetyltransferase (KAT), involved in histone acetylation and contributing to tumour recurrence, invasion and migration. A study reported that miR-N5 targets the 3’-UTR of CREBBP to suppress its expression, which in turn mediates H3K56 acetylation in the promoter regions of EGFR, β-catenin, and CDH1 in prostate cancer [42]. Consistently, another study demonstrated that low expression of CREBBP or EP300 inhibits H3K27 acetylation via the FBXW7–NOTCH–CCL2/CSF1 axis to induce the polarisation of tumour-associated macrophages to the M2 phenotype and tumour cell progression in diffuse large B-cell lymphoma (DLBCL) [43]. The results of this study are consistent with the abovementioned studies, indicating that CREBBP can regulate H3K27ac to affect the chromatin conformation of CCL20, resulting in increased expression of CCL20 (Fig. 8).

Furthermore, mechanisms underlying the regulation of Med15 expression in HCC were examined. Med15, a member of the mediator complex, was remarkably upregulated in HCC and correlated with tumour stage, histological grade and unfavourable clinical outcomes (Figs. S9 and 10). Med15 acts as a medium to connect with regulatory proteins and (Pol II), thereby modulating Pol II-induced transcription [26]. Several studies have reported that altered expression of Med15 contributes to tumorigenesis. Med15 is associated with worse survival rates in renal cell carcinoma (RCC), and its upregulation significantly increases tumour proliferation, migration and invasion [44]. In addition, Med15 is overexpressed in HCC [45]; however, its function remains unknown. In this study, the correlation between Med15 and HOXD3 was validated using TCGA database, RT-PCR, western blotting, Ch-IP and dual-luciferase reporter assays. The results demonstrated that HOXD3 induced the transcription of Med15, which triggered a change in the transcription of the CCL20 gene (Fig. 8). Therefore, the regulatory mechanisms of elevated CCL20 expression in HCC were further examined.

**Fig. 9:**
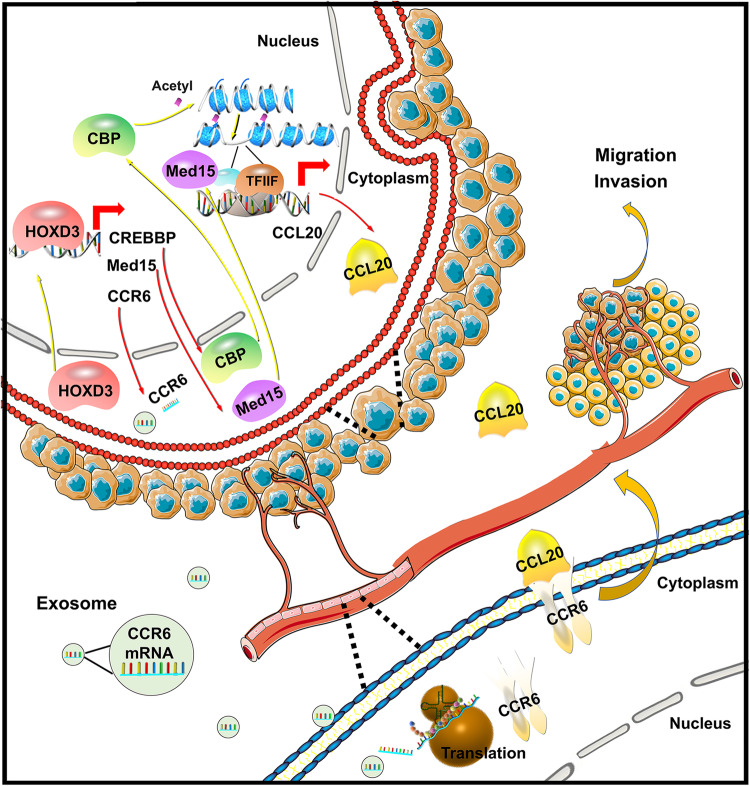
Molecular mechanism for the role of HOXD3-CREBBP/MED15-CCL20-CCR6 axis in HCC. HOXD3 could directly regulate the expressions of CREBBP and MED15. Both of them could activate H3K27 and Pol II, thereby modulating the CCL20 to promote metastasis, invasion and angiogenesis of HCC cells and EC cells. Furthermore, HOXD3 could induce the transcription of CCR6, which was delivered by HCC-HOXD3-exosomes from HCC cells to endothelial cells, thereby modulating angiogenesis by combining with CCL20.

In this study, exosomes derived from HOXD3-treated HCC cells participate in intercellular communication by delivering CCR6 mRNA, which regulates the angiogenesis of ECs and metastasis, invasion capability of HCC cells in vitro and vivo. In addition, HOXD3 directly modulates CREBBP and Med15, which subsequently enhances CCL20 expression by promoting H3K27 acetylation and induces Pol II-dependent transcription. The coordinated actions of CCR6 and CCL20 contribute to tumour progression by exerting multiple functions on both HCCs and ECs (Fig. 9). Therefore, this study revealed the function of the HOXD3–CREBBP/Med15–CCL20–CCR6 axis in regulating invasion and migration in HCC, thereby providing new therapeutic targets for HCC.

## Materials and methods

### Tissue specimens

Informed consent was collected from HCC patients, and ethical approval was obtained from the Ethics Committee of Xi’an Jiaotong University. HCC tissues and healthy adjacent tissues from patients were randomly obtained in the Second Affiliated Hospital of Xi’an Jiaotong University, China, from March 2020 to January 2021. Primary tumour samples were confirmed by pathological analysis in the Department of Pathology of The Second Affiliated Hospital of Xi’an Jiaotong University.

### Cell culture and transfection

Two human HCC cell lines (MHCC-97H and Huh7) and normal human liver cells (HL-7702) used in this study were purchased from Shanghai Zhong Qiao Xin Zhou Biotechnology Co., Ltd. All cell lines were tested and authenticated by their manufacturers. All cells were grown in DMEM (Basalmedia, Shanghai, China) containing 10% foetal bovine serum (Biological Industries, Cromwell, CT, USA), and HUVECs were grown in endothelial cell medium (ECM) containing basal medium 500 mL, FBS 25 mL, endothelial cell growth supplement 5 mL, and penicillin/streptomycin 5 mL at 37 °C in a humidified 5% CO_2_. The Universal Mycoplasma Detection Kit (ATCC 30–1012 K) was used to detect mycoplasma contamination before using the cells for further analysis, and the cells were found to be free of contamination.

Knockdown and overexpression experiments were conducted to elucidate the functional impact of HOXD3, CCR6, and CCL20 genes on HCC cancer. Lentiviral vectors pLKO.1-EGFP-Puro were utilised for cloning shRNAs targeting HOXD3, CCR6, and CCL20, along with a non-specific scramble shRNA sequence as an NC. Subsequently, pLKO.1-shHOXD3, CCR6 and CCL20 constructs were co-transfected with Pspax2 and pMD2.G lentiviral packaging plasmids into HEK293T cells to generate lentivirus. The lentivirus supernatant was collected and added to the culture medium of HCC cells for shRNA transduction. Furthermore, HOXD3, CCR6, CREBBP, and CCL20 were cloned into pcDNA3.1-EGFP vectors, and transfection was performed using Lipofectamine 2000 (Invitrogen).

### Cell co-culture

The HCC cell lines (MHCC-97H and Huh7) that had received treatment were subjected to co-culture with HUVECs at a ratio of 1:5. The co-cultured cells were maintained in a mixture of ECM and DMEM at a 1:1 ratio, and incubated at 37 °C in a humidified 5% CO_2_ environment.

### RNA extraction and quantitative real-time PCR assay

Total RNA was extracted from tissues and cell lines using TRIzoL (Invitrogen, California, USA) according to the manufacturer’s instructions, and reverse transcription was performed using a cDNA Synthesis Kit (Yeasen, Shanghai, China) according to the manufacturer’s instructions. qRT-PCR was carried out using the SYBR Green PCR kit (Yeasen, Shanghai, China) according to a method described previously [46]. The primers are listed in Table S1. Each experiment was repeated three independent times. The relative fold change in RNA expression was tested using the 2^−ΔΔCt^ method.

### Protein extraction and western blotting

Western blotting was performed as described previously [47]. Briefly, all tissue samples and HCC cells were lysed with RIPA buffer (Pioneer, Xi’an, China) containing protease inhibitors (Roche, Indianapolis, IN, USA). Protein concentration was quantified by a BCA Protein Assay Kit (Thermo Fisher Scientific, USA). Equal amounts of protein lysates were separated on 10–12% sodium dodecyl sulphate–polyacrylamide gels, Then, the proteins were transferred onto polyvinylidene difluoride membranes (Merck Millipore, Germany). After blocked in 5% skim milk 1 h at room temperature, the membranes were incubated with specific primary antibodies at 4 °C overnight. The following day, the membranes were washed thrice with TBST and incubated with the appropriate secondary antibody for 1 h. Protein bands were visualised using an enhanced chemiluminescence detection kit (Fdbio, Hangzhou, China). Imaging signals were acquired and analysed on ChemiDoc™ Touch (Bio-Rad, United States). The antibodies are listed in Table S2.

### Chromatin immunoprecipitation assay

Chromatin immunoprecipitation (ChIP) assay was performed as previously described [48]. Briefly, cells were crosslinked in 1% formaldehyde for 15 min at 37 °C and subsequently incubated with glycine (0.125 mol/L) for 30 min to stop crosslinking. Chromatin was fragmented via sonication, and the lysate was immunoprecipitated using Dynabeads (Invitrogen, Carlsbad, CA, USA) and antibodies against HOXD3, H3K27ac, PoII and IgG at 4 °C overnight. After DNA elution and purification, qRT-PCR was performed using IgG as a negative control. The primer sequences used are listed in Table S1, and the antibodies used are listed in Table S2.

### Dual-luciferase reporter assay

The promoter regions of CCR6, CREBBP and Med15, which contain the activating region, were cloned into the pGL3-basic vector (Promega, Madison, WI, USA). These constructs were subsequently co-transfected with HOXD3-Ctrl and HOXD3 in Huh7 and MHCC-97H cells, respectively. The Dual-Luciferase Reporter assay system (Promega) was employed to assess luciferase activity.

### Co-immunoprecipitation

CO-IP assays were performed to measure the relationship between two proteins as previously described [16]. Briefly, Cells were harvested using RIPA buffer, and the lysate was incubated overnight with a primary antibody against CBP. The protein–antibody complex was incubated with Dynabeads for 2 h and boiled with the loading buffer for 10 min. The obtained supernatant was used for western blotting.

### Wound healing assay

HCCs were seeded on 6-well plates and transfected after reaching 50% confluence. A scratch was made with a sterile 10-µl pipette tip. The cellular debris was removed by PBS, and cells were cultured in a fresh medium with 1% FBS at 37 °C with 5% CO_2_. Wound areas were captured at 0, 48 and 72 h using a microscope.

### Transwell invasion assay

The invasion of cells was tested using transwell chambers with a porous polycarbonate filter. Approximately 2.0 × 10^4^ cells in 200 μL of serum-free medium were incubated in the upper chamber, which was coated with Matrigel (Becton Dickinson Biosciences, MA, USA), whereas 600 μL of 10% FBS was added to the lower chamber. After 48 h of incubation, the cells on the upper surface were removed. The invasive cells on the lower surface were stained with 0.5% crystal violet and photographed.

### Capillary tube formation assay

The tube formation assay was performed as described previously [49]. Briefly, transfected or exosomes treated HUVECs (4 × 10^4^ cells/well) were plated on 50 μL of Matrigel (Becton Dickinson Biosciences, MA, USA) in 96-well and for 8 h to test the formation of vessel-like structures. Tube formation was observed and imaged with a microscope.

### Spheroid sprouting assay

Spheroids sprouting assay as described previously [8]. Briefly, spheroids were embedded into a collagen gel and allowed to polymerise in an incubator at 37 °C for 1 h, followed by the addition of a growth medium. After 72 h, the spheroids formed sprouts, which were imaged using a Leica microscope at 4× and 10× magnification.

### Immunohistochemical analysis

Immunohistochemical (IHC) analysis was performed to examine the expression of genes in HCC and normal tumour tissues at the protein level. It was performed according to standard protocols as described previously [46].

### Immunofluorescence microscopy

Cells were seeded into Nunc Glass Bottom Dishes (Thermo Fisher Scientific, Waltham, MA, USA). After 48 h of transfection, the cells were fixed with 4% formaldehyde, washed with PBST, and blocked with BSA for 1 h. The cells were incubated overnight at 4 °C with rabbit anti-CREBBP and mouse anti-H3K27 antibodies. Secondary antibodies stained with 4’,6-diamidino-2-phenylindole (DAPI) were used for detection. The images were captured using a fluorescence microscope (Nikon, Tokyo, Japan).

### Exosome isolation

The HOXD3 vector and control vector (HOXD3-Ctrl) were transfected into Huh7 and MHCC-97H cell lines, respectively. After 48 h, the culture supernatants were collected. The collected supernatants underwent differential centrifugation at 800 ×g for 5 min and 2,000 ×g for 10 min. The resulting supernatant was filtered and further ultra-centrifuged at 110,000×*g* for 2 h (repeated three times) at 4 °C. The exosomes were then collected from the pellet and resuspended in PBS. These exosomes were designated as Huh7-HOXD3-exosomes, Huh7-HOXD3-Ctrl-exosomes, MHCC-97H-HOXD3-exosomes and MHCC-97H-HOXD3-Ctrl-exosomes, respectively. The exosome particle size and morphology were examined using transmission electron microscopy (TEM), while their markers and concentration were identified through Western blotting assay and nanoparticle tracking analysis (NTA), respectively.

### Exosome cytoflex flow cytometer analysis

The exosomes (5–10 μl) were diluted using a phosphate-balanced solution at three concentration gradients of 1/10, 1/100, and 1/1000. The Cytoflex Flow Cytometer (Beckman, USA) was employed to determine the optimal dilution concentration, where the average particle number remained below 10,000. Subsequently, the sample was incubated with 1 μg of PE-anti-mouse/rat CD81 or APC-CD63 antibodies for 15 min at room temperature. Following the incubation, the expression of markers was detected using the Cytoflex Flow Cytometer.

### Exosome uptake analysis with PKH26 labelling

Freshly isolated exosomes were labelled with the PKH26 Red Fluorescent Cell Linker Kit (Sigma-Aldrich, St. Louis, MO, USA) according to the manufacturer’s instructions. The nuclei were stained with DAPI, and the uptake of exosomes by HCC cells was identified by using a fluorescence confocal microscope

### In vivo matrigel plug assay

Nude BALB/c mice (age 5 weeks) were subcutaneously injected with 300 μL of Matrigel Mix and 150 μL of Huh7-HOXD3-exosomes or Huh7-HOXD3-Ctrl-exosomes (4 ×10^6^). After 14 days, Matrigel plugs were harvested and analysed for vascularity based on histological characteristics.

### Analysis of tumour migration in nude mice

Huh7 cells were transfected with a lentiviral vector with luciferase and shCCL20. A Lentiviral vector with luciferase and shCCL20-Ctrl as a negative control. Stable cell lines with CCL20 knockdown and negative-control cells were established. Male nude mice (age 6 weeks, 18–20 g) were obtained from the Experimental Animal Centre of Xi’an Jiaotong University and maintained in a specific pathogen-free environment with free access to food and water and a 12-h light/dark cycle. The mice were injected with Huh7 cells transfected with shCCL20-Ctrl and shCCL20, treated with Huh7-HOXD3-Ctrl-exosomes (HOXD3-ctrl-induced Huh7-derived exosomes), Huh7-HOXD3-exosomes (HOXD3-induced Huh7-derived exosomes) and Huh7-HOXD3-exosomes+CCR6 inhibitor-1 through the tail vein (All nude mice were randomly assigned to different groups). Subsequently, the mice were anaesthetised with isoflurane/oxygen, and the IVIS Spectrum (Xenogen, Alameda, CA, USA) was used for in vivo bioluminescence imaging. In addition, the survival duration of mice was monitored. Seventy days later, all mice were sacrificed. The experiments involving animals were conducted in compliance with the Guide for the Care and Use of Laboratory Animals, following approval from the Ethics Committee of Xi’an Jiaotong University.

### Quantification and statistical analysis

Data were expressed as mean ± SEM. All analyses were performed using GraphPad Prism 6.0 software. Student’s *t*-test and one-way ANOVA were used to compare two groups or more groups according to the nature of the data. Kaplan–Meier survival analysis was used to determine the overall survival of different groups. *p*-Value of <0.05 was considered significant.

### Supplementary information

Supplemental materials

Checklist

## Data Availability

Any additional data will be provided upon reasonable request.
