# Supplementary material for: HOXD3 promotes the migration and angiogenesis of hepatocellular carcinoma via modifying hepatocellular carcinoma cells exosome-delivered CCR6 and regulating chromatin conformation of CCL20
Source: Cell Death Dis. 2024 Mar 16;15(3):221. doi: 10.1038/s41419-024-06593-x (PMC10944507; doi:10.1038/s41419-024-06593-x)
Supplement: Supplementary file 1 — Supplemental materials [file 41419_2024_6593_MOESM1_ESM.pdf]

Fig. S1

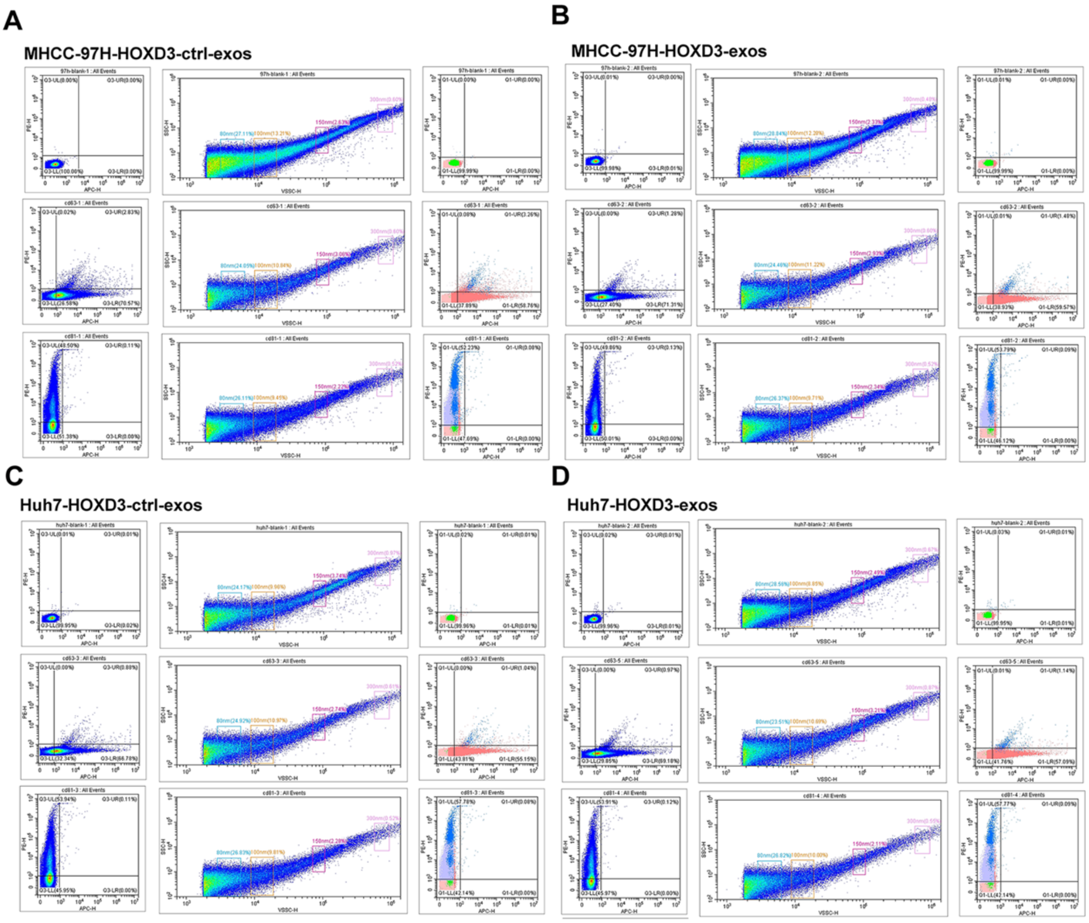
A
MHCC-97H-HOXD3-ctrl-exos
B
MHCC-97H-HOXD3-exos
C
Huh7-HOXD3-ctrl-exos
D
Huh7-HOXD3-exos

Fig. S2

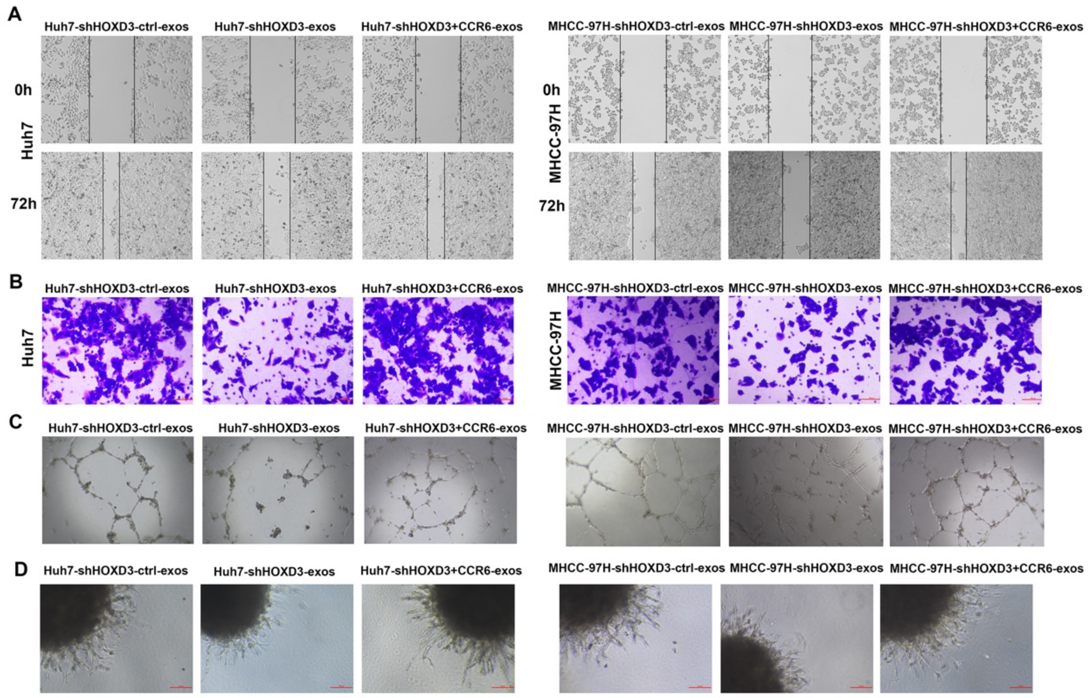
A
Huh7-shHOXD3-ctrl-exos
Huh7-shHOXD3-exos
Huh7-shHOXD3+CCR6-exos
MHCC-97H-shHOXD3-ctrl-exos
MHCC-97H-shHOXD3-exos
MHCC-97H-shHOXD3+CCR6-exos
0h
72h
Huh7
MHCC-97H
B
Huh7-shHOXD3-ctrl-exos
Huh7-shHOXD3-exos
Huh7-shHOXD3+CCR6-exos
MHCC-97H-shHOXD3-ctrl-exos
MHCC-97H-shHOXD3-exos
MHCC-97H-shHOXD3+CCR6-exos
Huh7
MHCC-97H
C
Huh7-shHOXD3-ctrl-exos
Huh7-shHOXD3-exos
Huh7-shHOXD3+CCR6-exos
MHCC-97H-shHOXD3-ctrl-exos
MHCC-97H-shHOXD3-exos
MHCC-97H-shHOXD3+CCR6-exos
D
Huh7-shHOXD3-ctrl-exos
Huh7-shHOXD3-exos
Huh7-shHOXD3+CCR6-exos
MHCC-97H-shHOXD3-ctrl-exos
MHCC-97H-shHOXD3-exos
MHCC-97H-shHOXD3+CCR6-exos

Fig. S3

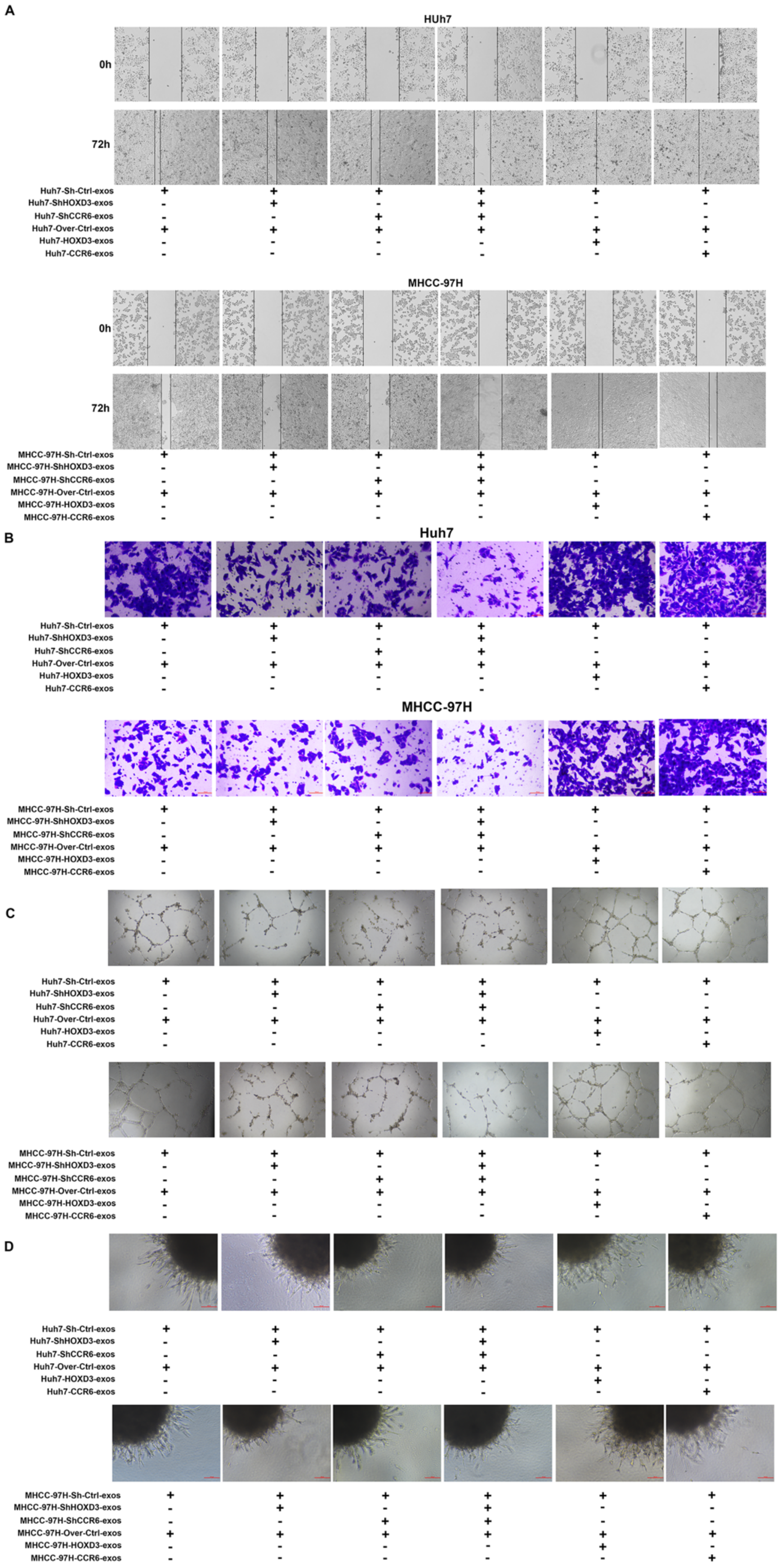

A
HUh7
0h
72h
Huh7-Sh-Ctrl-exos
Huh7-ShHOXD3-exos
Huh7-ShCCR6-exos
Huh7-Over-Ctrl-exos
Huh7-HOXD3-exos
Huh7-CCR6-exos
MHCC-97H
0h
72h
MHCC-97H-Sh-Ctrl-exos
MHCC-97H-ShHOXD3-exos
MHCC-97H-ShCCR6-exos
MHCC-97H-Over-Ctrl-exos
MHCC-97H-HOXD3-exos
MHCC-97H-CCR6-exos
B
Huh7
Huh7-Sh-Ctrl-exos
Huh7-ShHOXD3-exos
Huh7-ShCCR6-exos
Huh7-Over-Ctrl-exos
Huh7-HOXD3-exos
Huh7-CCR6-exos
MHCC-97H
MHCC-97H-Sh-Ctrl-exos
MHCC-97H-ShHOXD3-exos
MHCC-97H-ShCCR6-exos
MHCC-97H-Over-Ctrl-exos
MHCC-97H-HOXD3-exos
MHCC-97H-CCR6-exos
C
Huh7-Sh-Ctrl-exos
Huh7-ShHOXD3-exos
Huh7-ShCCR6-exos
Huh7-Over-Ctrl-exos
Huh7-HOXD3-exos
Huh7-CCR6-exos
MHCC-97H-Sh-Ctrl-exos
MHCC-97H-ShHOXD3-exos
MHCC-97H-ShCCR6-exos
MHCC-97H-Over-Ctrl-exos
MHCC-97H-HOXD3-exos
MHCC-97H-CCR6-exos
D
Huh7-Sh-Ctrl-exos
Huh7-ShHOXD3-exos
Huh7-ShCCR6-exos
Huh7-Over-Ctrl-exos
Huh7-HOXD3-exos
Huh7-CCR6-exos
MHCC-97H-Sh-Ctrl-exos
MHCC-97H-ShHOXD3-exos
MHCC-97H-ShCCR6-exos
MHCC-97H-Over-Ctrl-exos
MHCC-97H-HOXD3-exos
MHCC-97H-CCR6-exos

Fig. S4

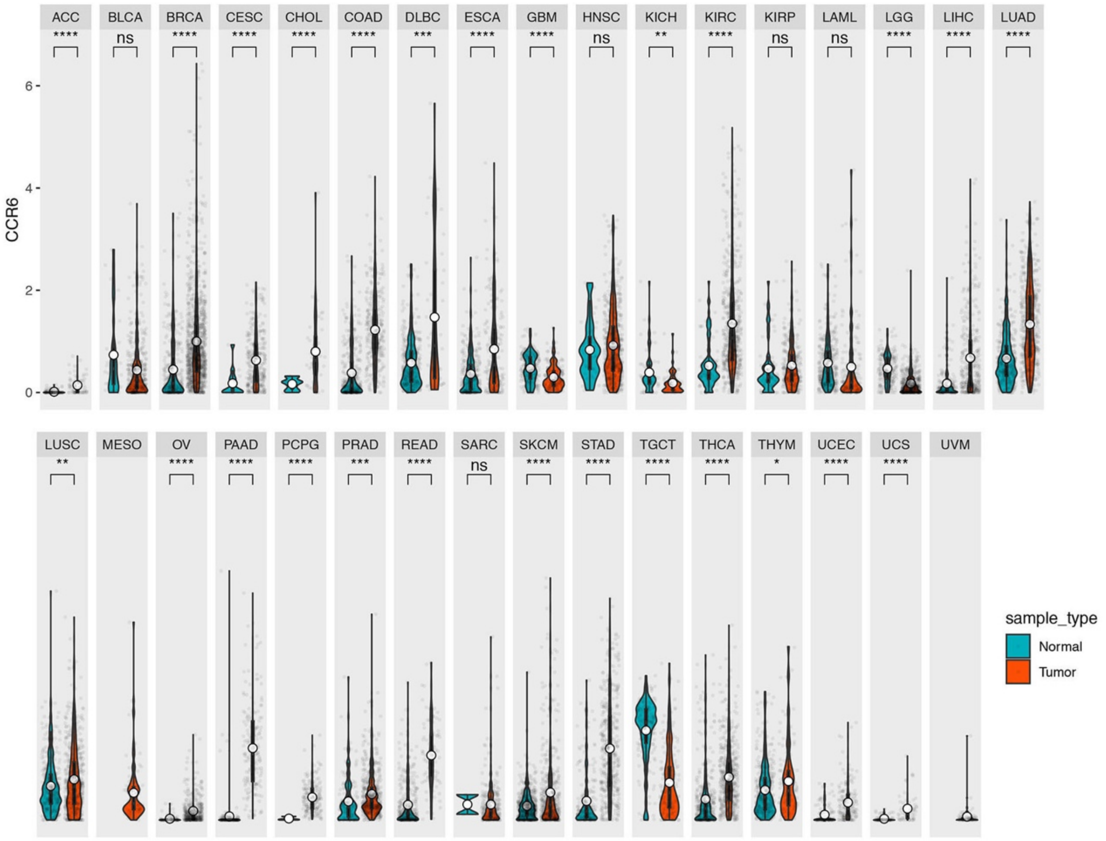
ACC
BLCA
BRCA
CESC
CHOL
COAD
DLBC
ESCA
GBM
HNSC
KICH
KIRC
KIRP
LAML
LGG
LIHC
LUAD
LUSC
MESO
OV
PAAD
PCPG
PRAD
READ
SARC
SKCM
STAD
TGCT
THCA
THYM
UCEC
UCS
UVM
CCR6
sample_type
Normal
Tumor

Fig. S5

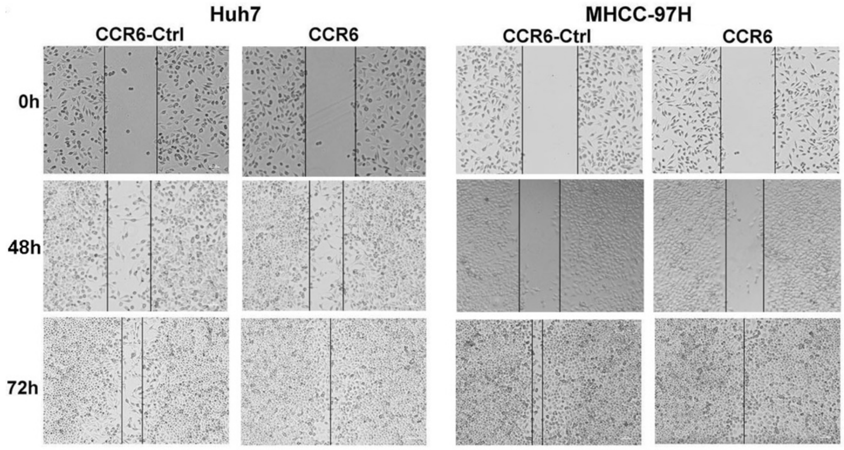
Huh7
MHCC-97H
CCR6-Ctrl
CCR6
CCR6-Ctrl
CCR6
0h
48h
72h

Fig. S6

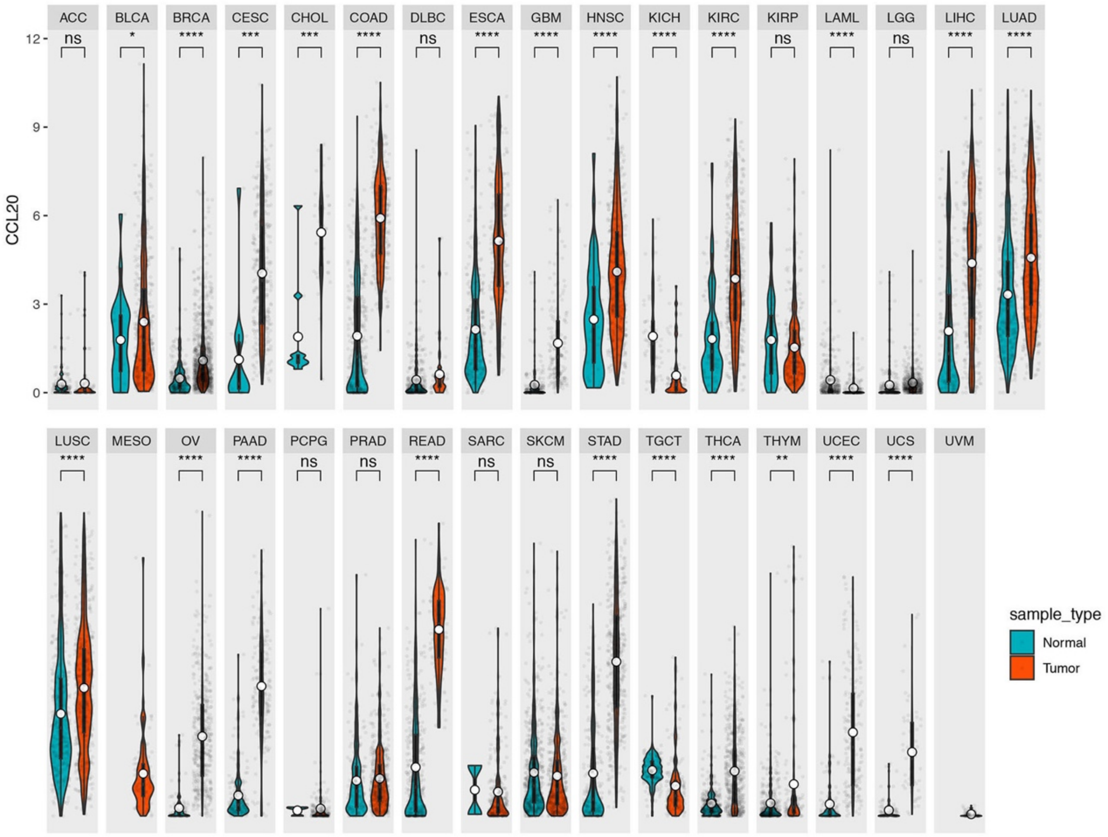
ACC
BLCA
BRCA
CESC
CHOL
COAD
DLBC
ESCA
GBM
HNSC
KICH
KIRC
KIRP
LAML
LGG
LIHC
LUAD
LUSC
MESO
OV
PAAD
PCPG
PRAD
READ
SARC
SKCM
STAD
TGCT
THCA
THYM
UCEC
UCS
UVM
CCL20
sample_type
Normal
Tumor

Fig. S7

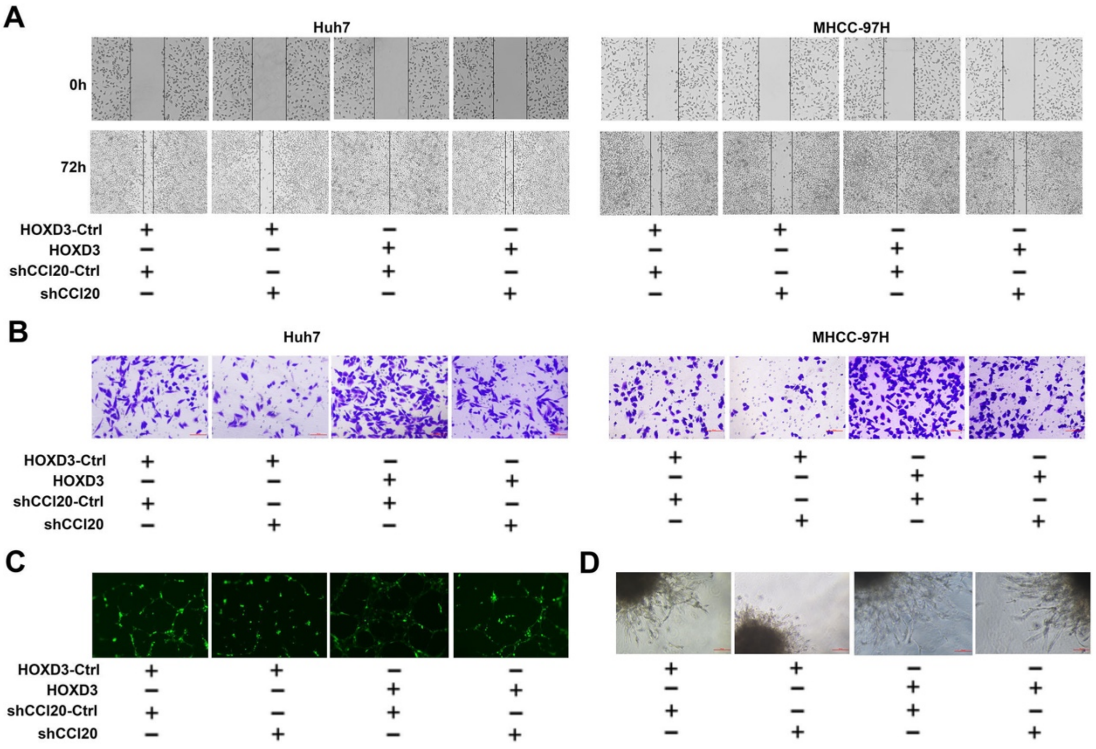
A
Huh7
MHCC-97H
0h
72h
HOXD3-Ctrl
HOXD3
shCCl20-Ctrl
shCCl20
B
Huh7
MHCC-97H
C
D

Fig. S8

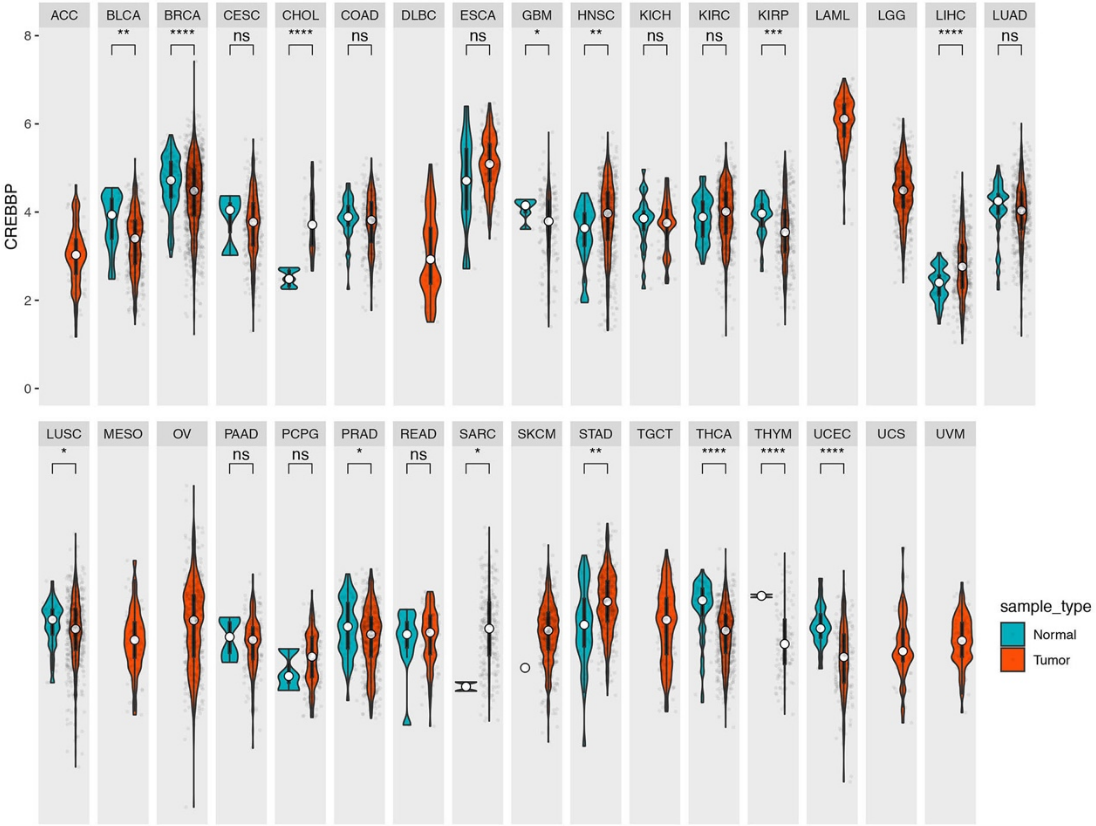
ACC
BLCA
BRCA
CESC
CHOL
COAD
DLBC
ESCA
GBM
HNSC
KICH
KIRC
KIRP
LAML
LGG
LIHC
LUAD
**
****
ns
****
ns
ns
*
**
ns
ns
***
****
ns
CREBBP
8
6
4
2
0
LUSC
MESO
OV
PAAD
PCPG
PRAD
READ
SARC
SKCM
STAD
TGCT
THCA
THYM
UCEC
UCS
UVM
*
ns
ns
*
ns
*
**
****
****
****
sample_type
Normal
Tumor

Fig. S9

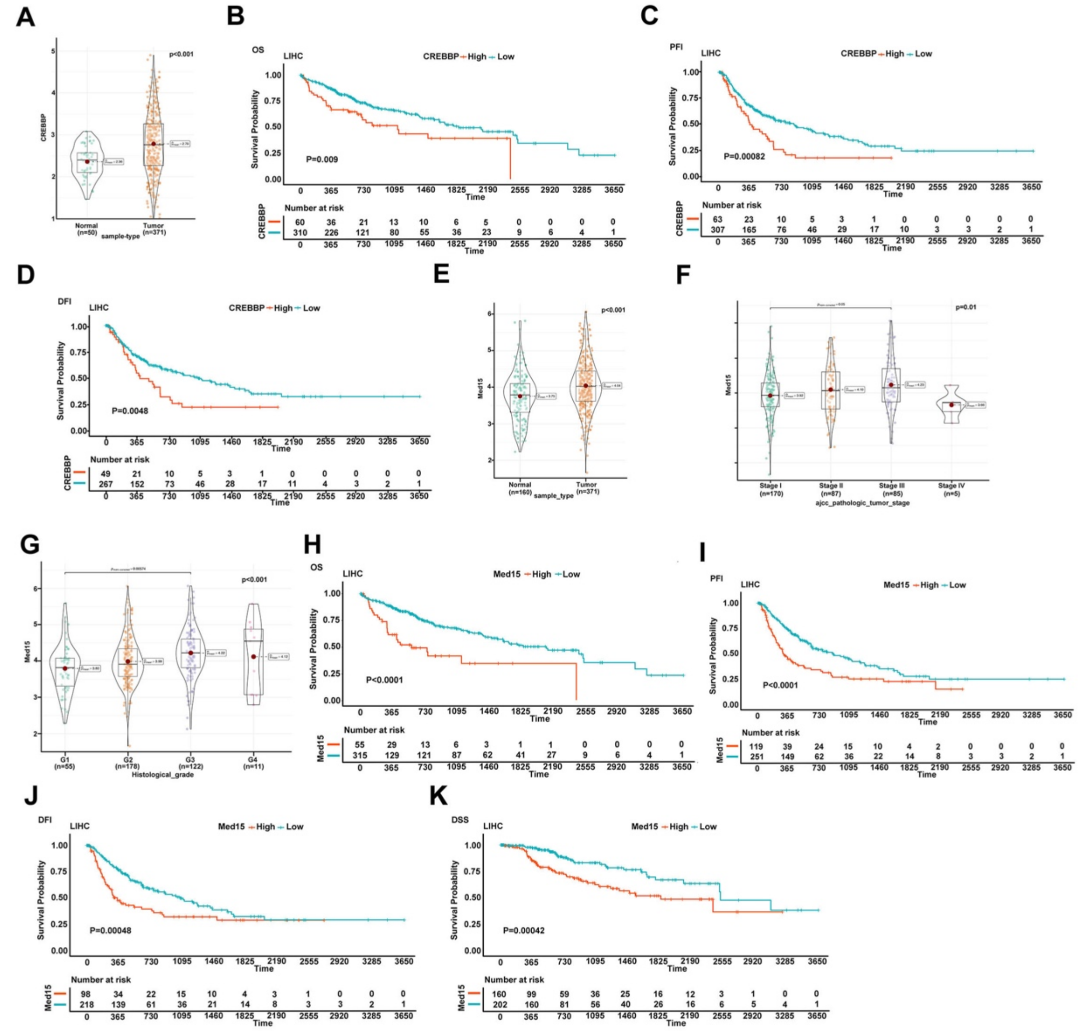

A
CREBBP
p<0.001
Normal (n=50)
Tumor (n=371)
sample-type
B
OS
LIHC
CREBBP High Low
Survival Probability
P=0.009
Time
Number at risk
CREBBP
60 36 21 13 10 6 5 0 0 0 0
310 226 121 80 55 36 23 9 6 4 1
0 365 730 1095 1460 1825 2190 2555 2920 3285 3650
C
PFI
LIHC
CREBBP High Low
Survival Probability
P=0.00082
Time
Number at risk
CREBBP
63 23 10 5 3 1 0 0 0 0 0
307 165 76 46 29 17 10 3 3 2 1
0 365 730 1095 1460 1825 2190 2555 2920 3285 3650
D
DFI
LIHC
CREBBP High Low
Survival Probability
P=0.0048
Time
Number at risk
CREBBP
49 21 10 5 3 1 0 0 0 0 0
267 152 73 46 28 17 11 4 3 2 1
0 365 730 1095 1460 1825 2190 2555 2920 3285 3650
E
Med15
p<0.001
Normal (n=160)
Tumor (n=371)
sample_type
F
Med15
p=0.01
Stage I (n=170)
Stage II (n=87)
Stage III (n=85)
Stage IV (n=5)
ajcc_pathologic_tumor_stage
G
Med15
p<0.001
G1 (n=55)
G2 (n=178)
G3 (n=122)
G4 (n=11)
Histological_grade
H
OS
LIHC
Med15 High Low
Survival Probability
P<0.0001
Time
Number at risk
Med15
55 29 13 6 3 1 1 0 0 0 0
315 129 121 87 62 41 27 9 6 4 1
0 365 730 1095 1460 1825 2190 2555 2920 3285 3650
I
PFI
LIHC
Med15 High Low
Survival Probability
P<0.0001
Time
Number at risk
Med15
119 39 24 15 10 4 2 0 0 0 0
251 149 62 36 22 14 8 3 3 2 1
0 365 730 1095 1460 1825 2190 2555 2920 3285 3650
J
DFI
LIHC
Med15 High Low
Survival Probability
P=0.00048
Time
Number at risk
Med15
98 34 22 15 10 4 3 1 0 0 0
218 139 61 36 21 14 8 3 3 2 1
0 365 730 1095 1460 1825 2190 2555 2920 3285 3650
K
DSS
LIHC
Med15 High Low
Survival Probability
P=0.00042
Time
Number at risk
Med15
160 99 59 36 25 16 12 3 1 0 0
202 160 81 56 40 26 16 6 5 4 1
0 365 730 1095 1460 1825 2190 2555 2920 3285 3650

Fig. S10

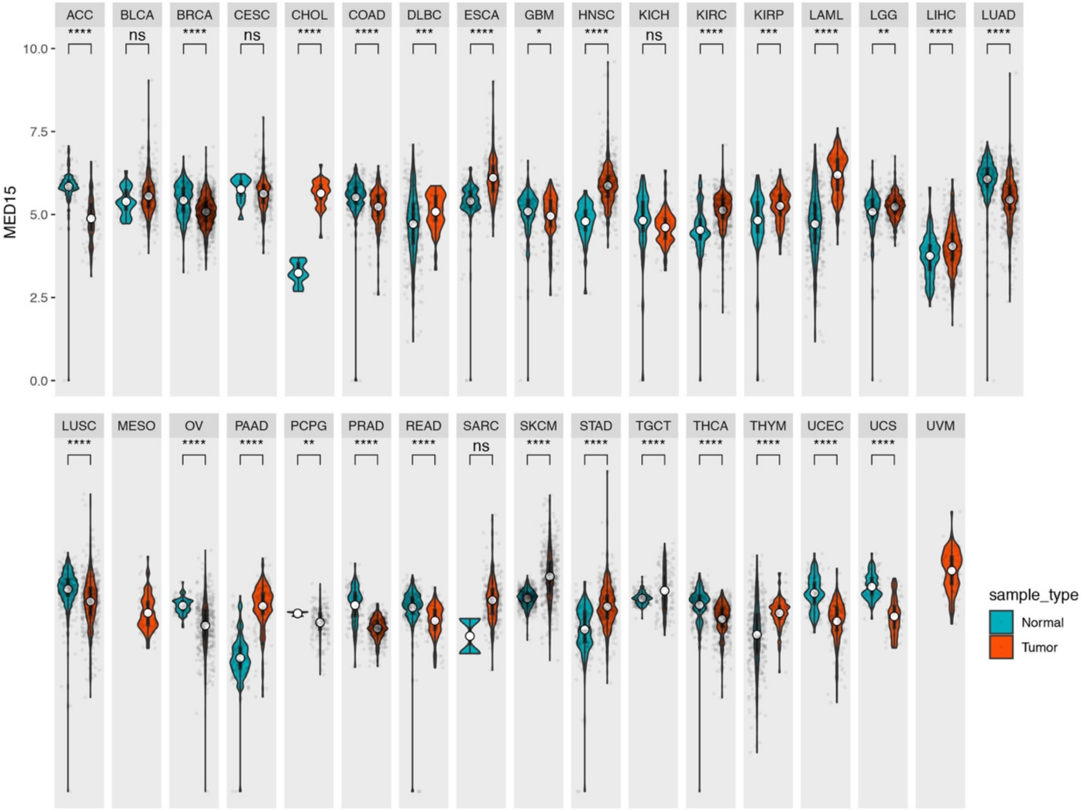

ACC
BLCA
BRCA
CESC
CHOL
COAD
DLBC
ESCA
GBM
HNSC
KICH
KIRC
KIRP
LAML
LGG
LIHC
LUAD
****
ns
****
ns
****
****
***
****
*
****
ns
****
***
****
**
****
****
10.0
7.5
5.0
2.5
0.0
MED15
LUSC
MESO
OV
PAAD
PCPG
PRAD
READ
SARC
SKCM
STAD
TGCT
THCA
THYM
UCEC
UCS
UVM
****
****
****
**
****
****
ns
****
****
****
****
****
****
****
sample_type
Normal
Tumor

Fig. S11

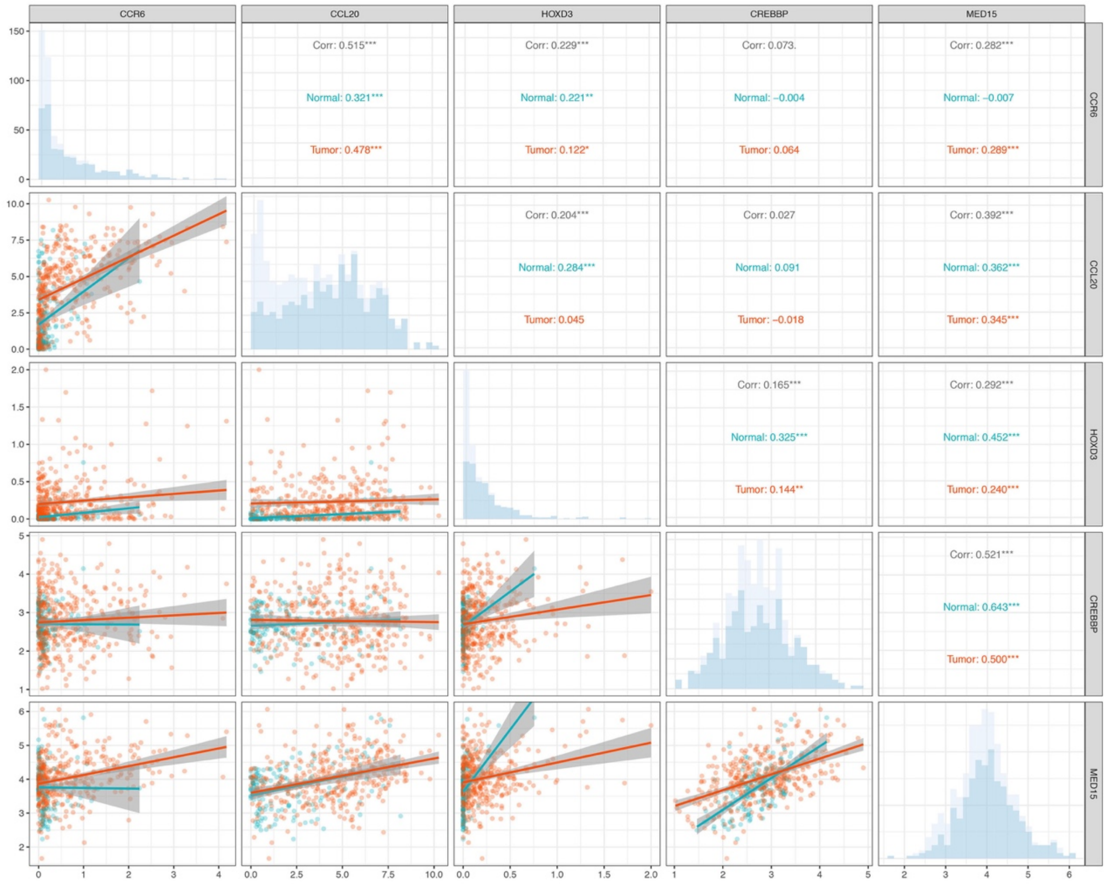
CCR6
CCL20
HOXD3
CREBBP
MED15
Corr: 0.515***
Normal: 0.321***
Tumor: 0.478***
Corr: 0.229***
Normal: 0.221**
Tumor: 0.122*
Corr: 0.073.
Normal: −0.004
Tumor: 0.064
Corr: 0.282***
Normal: −0.007
Tumor: 0.289***
Corr: 0.204***
Normal: 0.284***
Tumor: 0.045
Corr: 0.027
Normal: 0.091
Tumor: −0.018
Corr: 0.392***
Normal: 0.362***
Tumor: 0.345***
Corr: 0.165***
Normal: 0.325***
Tumor: 0.144**
Corr: 0.292***
Normal: 0.452***
Tumor: 0.240***
Corr: 0.521***
Normal: 0.643***
Tumor: 0.500***

Fig. S12

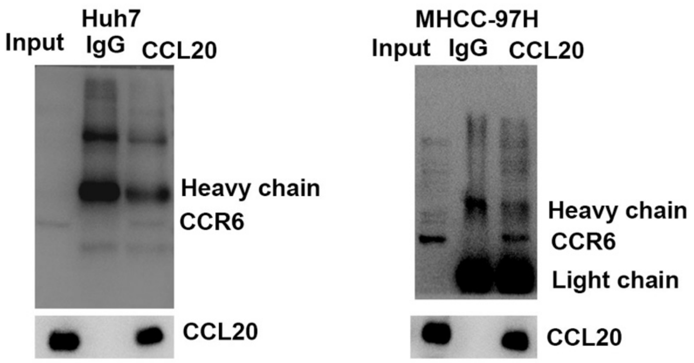

Fig. S13

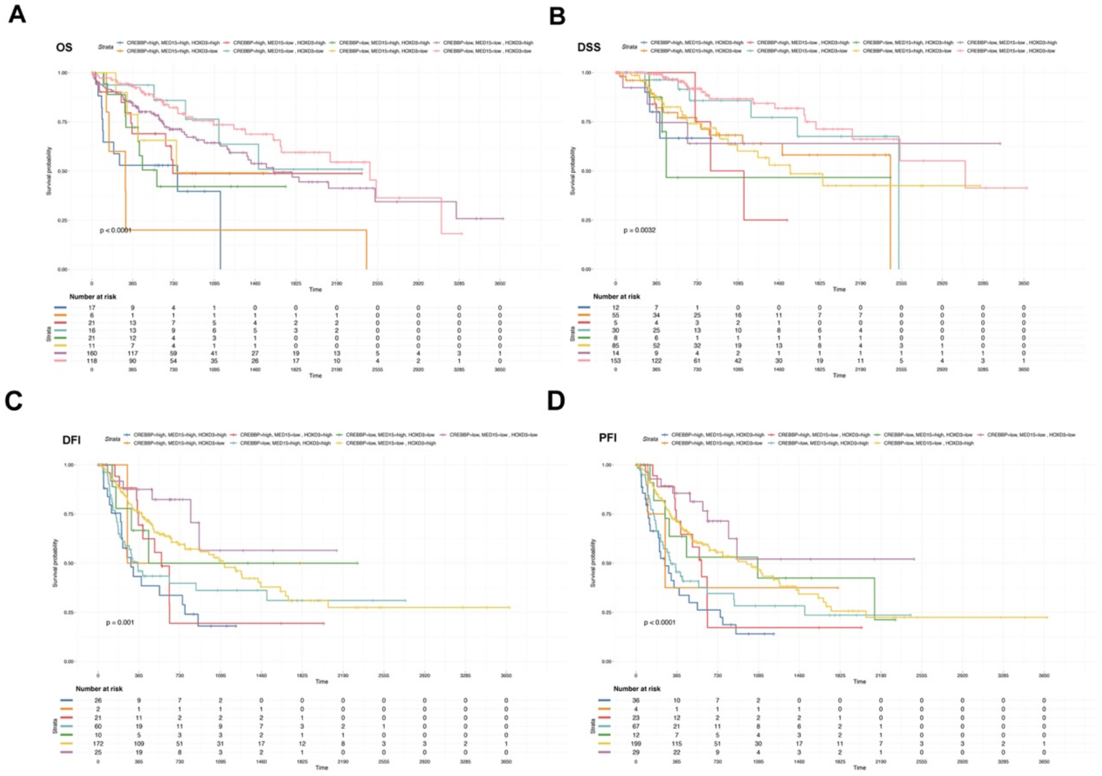

Supplementary Figure legends

Fig. S1 Cytoflex Flow Cytometer analysis of the exosomes marked with CD63 and CD81.

Fig. S2 Overexpression of CCR6 reverses the function of shHOXD3-exos in the metastasis and invasion of HCC cells. A-D Wound healing, transwell invasion, tube formation and 3D spheroid sprouting assays revealed that CCR6 overexpression reversed the role of shHOXD3 in the metastasis, invasion, and angiogenesis capability of HCC cells and ECs (scale bar = 100 μm).

Fig. S3 The function of HOXD3 and CCR6-exos in the metastasis, invasion and angiogenesis of HCC cells and ECs. A-D Wound healing, transwell invasion, tube formation and 3D spheroid sprouting assays revealed that the role of HOXD3 and CCR6-exos in the metastasis, invasion, and angiogenesis capability of HCC cells and ECs (scale bar =100 μm).

Fig. S4 The expression of CCR6 in various cancers in the TCGA and GTEX databases.

Fig. S5 Scratch wound healing assay was performed to identify the metastasis of HCC cells at 0, 48, and 72h. HCC cells were treated with the CCR6, CCR6-Ctrl (scale bar = 100 μm).

Fig. S6 The expression of CCL20 in various cancers in the TCGA and GTEX databases.

Fig. S7 Inhibition of CCL20 reverses the stimulatory function of HOXD3 in the metastasis, invasion and angiogenesis of HCC cells and ECs. A-D Wound healing, transwell invasion, tube formation and 3D spheroid sprouting assays revealed that CCL20 downregulation reversed the stimulatory role of HOXD3 in the metastasis, invasion, and angiogenesis capability of HCC cells and ECs (scale bar = 100 μm).

Fig. S8 The expression of CREBBP in various cancers in the TCGA database.

Fig. S9 Association with CREBBP and Med15 expression and clinicopathological characteristics. A Increased CREBBP of HCC tissues compared with normal tissues in the TCGA and GTEx database. **B-D** Survival curves of OS, PFI, and DFI between CREBBP-high and -low patients with HCC. **E** Increased Med15 of HCC tissues compared with normal tissues in the TCGA and GTEx database. **F** Pathologic stage **G** Histological grade in HCC patients in TCGA cohort. **H-K** Survival curves of OS, PFI, DFI, and DSS between Med15-high and -low patients with HCC.

Fig. S10 The expression of Med15 in various cancers in the TCGA and GTEX databases.

Fig. S11 The correlation of the HOXD3, CREBBP, Med15, CCL20, and CCR6 in the HCC.

Fig. S12 The CCL20 combines with the CCR6 in HCC cells.

Fig. S13 Kaplan-Meier survival curves comparing the co-high and low expression of HOXD3, CREBBP, and Med15 in HCC. A–D Survival curves of OS, DSS, DFI, and PFI between HOXD3, CREBBP, and Med15-co-high and -low patients with HCC.

Western blotting results

Figure 6

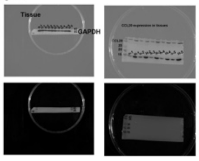

Figure 3

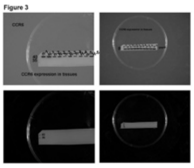

Figure 1

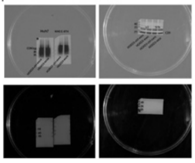

Figure 4

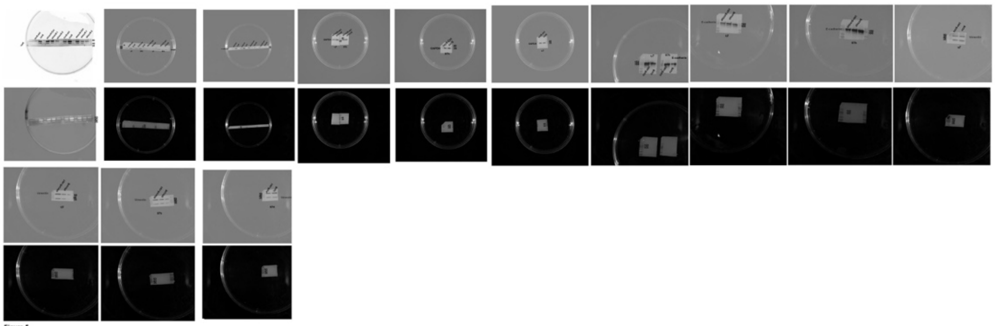

Figure 5

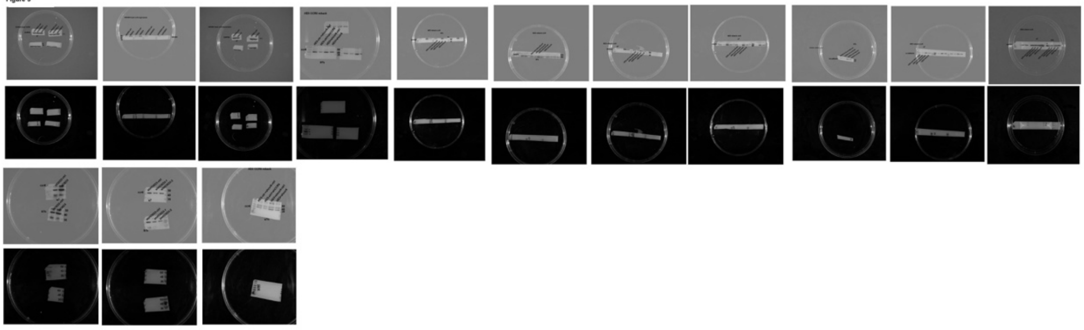

Figure 7

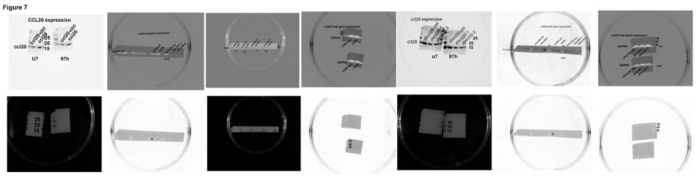

Figure 8

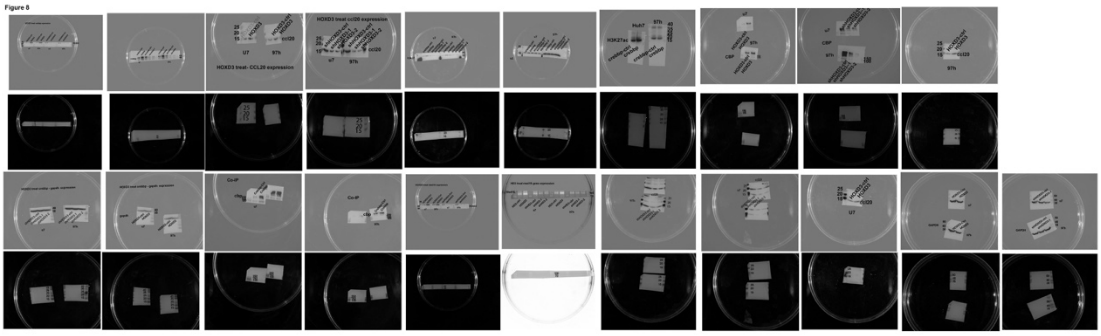

Supplementary Table 1.

Primers and oligonucleotides used in this work.

Name	Sequence(5’-3’)
GAPDH-F	5′-GGAGCGAGATCCCTCCAAAAT-3′
GAPDH-R	5′-GGCTGTTGTCATACTTCTCATGG-3′
CREBBP-F	5′-ACAAGCGAAACCAACAAACC-3′
CREBBP-R	5′-AAAGAAGTGGCATTCTGTTGC-3′
Med15-F	5′-AACTCCAACGTCAGCTCTGG-3′
Med15-R	5′-GTTGAAGACAGGTGAGCGGA-3′
CCR6-F	5′-TGCGTGGGTTTTCAGCAATG-3′
CCR6-R	5′-CCAAATTTGCAGCCGTCACA-3′
CCL20-F	5′-GTGCTGCTACTCCACCTCTG-3′
CCL20-R	5′-TGTTGCTTGCAAGAAAGTCCA-3′
CHIP-PCR CCR6-S-1	5′-TGGGAAGTTACTCAACGCGT-3′
CHIP-PCR CCR6-A-1	5′-TCCACCCCTCTCCTTCCTTT-3′
CHIP-PCR CREBBP-S-1	5′-GTGAGGACATGGGTTCCAGG-3′
CHIP-PCR CREBBP-A-1	5′-AGCTTGAGCCATTCCCTGAC-3′
CHIP-PCR Med15-S-1	5′-TCGTTTGACATCTGGCCTCC-3′
CHIP-PCR Med15-A-1	5′-AGAGCGACCAGATGGACTCT-3′
CHIP-PCR CCL20-S-1	5′-TGCGGGTTTTTTTATGATG-3′
CHIP-PCR CCL20-A-1	5′-TATAGCAAATATTGGGAATG-3′
shCCL20-F	5'-CCGGAGTTGTCTGTGTGCGCAAATCCTCGAGGATTTG CGCACACAGACAACTTTTTG-3'
shCCL20-R	5'-AATTCAAAAAAGTTGTCTGTGTGCGCAAATCCTCGAG GATTTGCGCACACAGACAACT-3'
shCCR6-F	5'-CCGGTCGACTCCAGTGAAGATTATTCTCGAGAATAAT CTTCACTGGAGTCGATTTTTG-3'
shCCR6-R	5'-AATTCAAAAATCGACTCCAGTGAAGATTATTCTCGAG AATAATCTTCACTGGAGTCGA-3'
shHOXD3-F	5'-CCGGGAGTCTCGACAGAACTCCACTCGAGTGGAGTT CTGTCGAGACTCTTTTTG-3'
shHOXD3-R	5'-AATTCAAAAAGAGTCTCGACAGAACTCCACTCGAGT GGAGTTCTGTCGAGACTC-3'
siCREBBP-S1	5'-CGCAGGUUUCCCCGCAAAU-3'
siCREBBP-A1	5'-AUUUGCGGGGAAACCUGCG-3'
siCREBBP-S2	5'-AGUUACUAUUGAGGAGGCCTT-3'
siCREBBP-A2	5'-GGCCUCCUCAAUAGUAACUTT-3'

Supplementary Table 2.

Antibody used in this work.

Name	Information
HOXD3	Santa Cruz Biotechnology, sc-130378
CCR6	ZEN BIO, 861520
N-cadherin	Cell Signaling technology, #14215
MMP9	Cell Signaling technology, #13667
CREBBP	Cell Signaling technology, #7389
E-cadherin	Cell Signaling technology, #14472
Vimentin	Cell Signaling technology, #46173
Med15	Proteintech Group, 11566-1-AP
CCL20	ABclonal, A1756(WB). Proteintech Group, 26527-1-AP(IHC)
CD31	ZEN BIO, M50921
H3K27	ACTIVE MOTIF, 39085
H3	Proteintech Group, 17168-1-AP
Pol II	Abcam, ab817
GAPDH	Abways technology, AB0038
Goat Anti-Rabbit IgG (H+L) HRP	Abways technology, AB0101
Goat Anti-Mouse IgG (H+L) HRP	Abways technology, AB0102
Flag	Proteintech Group, 20543-1-AP
IgG	Proteintech Group, 30000-0-AP
